# Synthesis of a New Class of *β*-Carbonyl Selenides Functionalized with Ester Groups with Antioxidant and Anticancer Properties—Part II

**DOI:** 10.3390/molecules29122866

**Published:** 2024-06-16

**Authors:** Anna Laskowska, Agata J. Pacuła-Miszewska, Magdalena Obieziurska-Fabisiak, Aneta Jastrzębska, Angelika Długosz-Pokorska, Katarzyna Gach-Janczak, Jacek Ścianowski

**Affiliations:** 1 Department of Organic Chemistry, Faculty of Chemistry, Nicolaus Copernicus University, 7 Gagarin Street, 87-100 Torun, Poland; annlas@doktorant.umk.pl (A.L.); pacula@umk.pl (A.J.P.-M.); magdao@umk.pl (M.O.-F.); 2 Department of Analytical Chemistry and Applied Spectroscopy, Faculty of Chemistry, Nicolaus Copernicus University in Torun, 7 Gagarin Street, 87-100 Torun, Poland; aj@umk.pl; 3 Department of Biomolecular Chemistry, Faculty of Medicine, Medical University of Lodz, 6/8 Mazowiecka Street, 92-215 Lodz, Poland; angelika.dlugosz@umed.lodz.pl (A.D.-P.); katarzyna.gach@umed.lodz.pl (K.G.-J.)

**Keywords:** 2-((2-oxopropyl)selanyl)benzoates, pharmacophore, antioxidant activity, antiproliferative activity

## Abstract

A series of phenyl *β*-carbonyl selenides with o-ester functionality substituted on the oxygen atom with chiral and achiral alkyl groups was synthesized. All compounds are the first examples of this type of organoselenium derivatives with an ester substituent in the ortho position. The obtained derivatives were tested as antioxidants and anticancer agents to see the influence of an ester functionality on the bioactivity of *β*-carbonyl selenides by replacing the *o*-amide group with an *o*-ester group. The best results as an antioxidant agent were observed for *O*-((1*R*,2*S*,5*R*)-(−)-2-isopropyl-5-methylcyclohexyl)-2-((2-oxopropyl)selanyl)benzoate. The most cytotoxic derivative against breast cancer MCF-7 cell lines was *O*-(methyl)-2-((2-oxopropyl)selanyl)benzoate and against human promyelocytic leukemia HL-60 was *O*-(2-pentyl)-2-((2-oxopropyl)selanyl)benzoate.

## 1. Introduction

**Figure 1:**
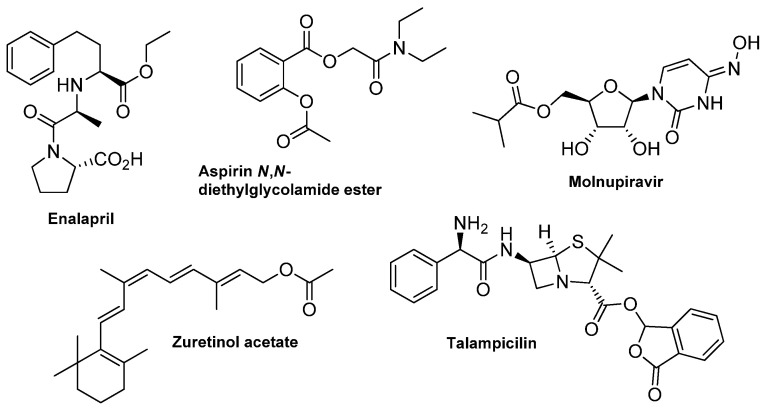
Examples of ester-type drugs.

Organic molecules, both of a synthetic and natural origin, are utilized in almost every field of industry, depending on their applicability, determined mainly by the particular functional groups incorporated into their structure. Among those, esters are one of the most exploited compounds, commonly known for their characteristic fragrance properties and widely used in the perfume industry [1]. In addition to the natural presence in essential oils and pheromones, the ester bond is also a dominating functionality in many primary (e.g., lipids and carbohydrates [2]) and secondary (e.g., lactones, terpenoids and steroids [3]) metabolites [4]. The ester linkage is essential for several biochemical pathways due to its ability and facile cleavage by esterases, hydrolase-type enzymes that split the ester into a carboxylic acid and alcohol [5,6,7]. In the context of drug development, the introduction of an ester moiety bears several advantages. The transformation of bioactive compounds like polar carboxyl or hydroxyl groups into an ester prodrug improves its solubility and bioavailability [8]. Increased membrane permeability facilitates the delivery and installation inside the cells, where the molecule can be hydrolyzed to its water-soluble active form [9,10]. Using esterification in drug modification solves the bioavailability problem of many known bioactive agents. Examples of drugs applied in their ester form are presented in Figure 1 [11,12,13].

**Scheme 1:**
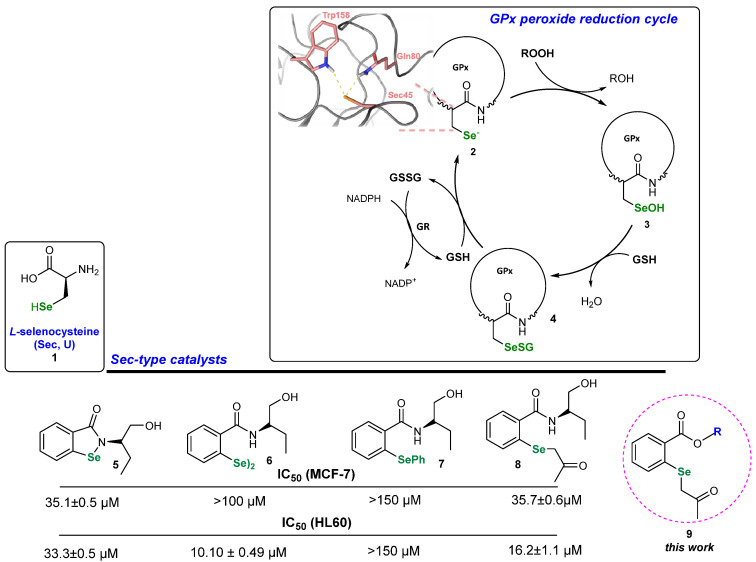
GPx activity cycle and examples of Sec-type catalysts **5**–**9**.

Organoselenium compounds like selenides, diselenides and selenols are well known to possess a multitude of possible biological activities due to the ability to mimic the activity of several selenoenzymes, including the antioxidant enzyme glutathione peroxidase (GPx), and they are involved in thyroid physiology, thioredoxin reductase (TRx) and iodothyronine deiodinase (ID) [14,15,16]. A catalytic reduction in peroxides by GPx is presented in Scheme 1 [17]. *L*-selenocysteine **1** builds up the active side of the enzyme and catalyzes the ROOH reduction to ROH [18]. The Se atom of **2** is maintained in its active ionized form due to the specific amino acid environment—glutamine and tryptophan—creating the catalytic triad with *L*-Sec [19]. The formed seleninic acid **3** is reduced to the starting RSe^−^ in a two-step process by two glutathione (GHS) molecules. Se-derivatives acting as artificial *L*-Sec can exhibit various bioactivities [20]. Until now, we have synthesized a diversified series of GPx mimetics, including benzisoselenazolones **5**, corresponding diselenides **6** [21] and phenyl selenides **7** [22]. An evaluation of their antiproliferative activity revealed that installing a -SePh functionality significantly decreases the reactivity. To address this issue, we created a new type of selenide-type catalyst possessing a 2-(2-oxopropyl)selanyl group **8** and showed that this modification significantly enhanced the reactivity of the tested compounds [23].

Herein, we wanted to examine the influence of an ester functionality on the bioactivity of *β*-carbonyl selenides by replacing the *o*-amide group with an *o*-ester substituent. For this purpose, we synthesized the first *β*-carbonyl phenyl selenides possessing an *o*-ester group and evaluated if this modification changes their antioxidant and anticancer properties. 

## 2. Results and Discussion

The first step in the research involved synthesizing a new class of compounds—*O*-substituted 2-((2-oxopropyl)selanyl)-benzoate derivatives. The procedure was based on the reaction of acetylated selenide **11**, formed in situ from 2-(chloroseleno)-benzoyl chloride **A** and acetone, with commercially available alcohols. Initially, the reaction of acetone with sodium bicarbonate yielded a carbanion which was used to acylate the selenium atom. A subsequent reaction with alcohol **B** furnished the final product **10** with only a 16% yield (entry 1, Table 1). In the case of a reaction with several other alcohols, the product was not formed. In the next attempt, NaHCO_3_ was replaced with triethylamine. In this way, the reaction efficiency increased to 54% (entry 2, Table 1). The reaction’s key step was mixing the ketone with triethylamine to form the carbanion and remove the hydrogen chloride produced in the reaction.

**Scheme 2:**
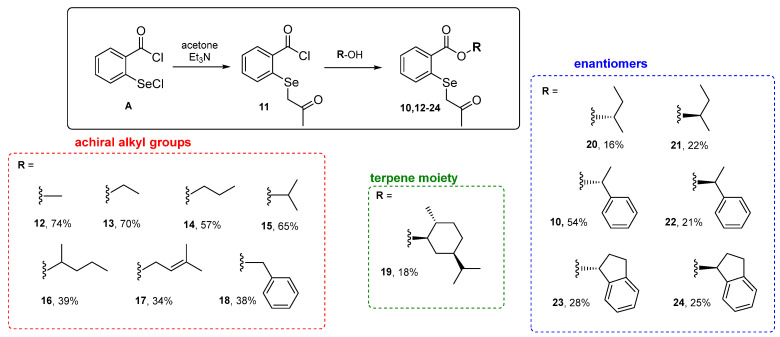
Synthetized *β*-carbonyl selenides with *o*-ester groups **10** and **12**–**24.**

The selected conditions (entry 2, Table 1) were applied in further reactions with commercially available alkyl achiral and chiral alcohols, including monocyclic terpene alcohol. A series of *β*-carbonyl selenides, seven alkyl derivatives **12**–**18** and seven optically active compounds, including *O*-terpenyl selenide **19** and three pairs of enantiomers **10** and **20**–**24**, were obtained with good yields (Scheme 2).

All derivatives were evaluated as antioxidants by two assays. Firstly, the NMR-based method was used, as presented by Iwaoka and co-workers [24]. The ability to reduce hydrogen peroxide was measured by the rate of dithiothreitol oxidation to disulphide (DTTred to DTTox). The active Se-catalyst is formed through the oxidation of selenide **25** to selenoxide **26** by hydrogen peroxide. Dithiothreitol (DTTred) reduces compound **26** to the starting selenide **25**. The substrate (DTTred) disappearance rate was estimated by recording changes in the 1H NMR spectrum in specific time intervals. The reaction equation and antioxidant test results (mean value ± standard deviation) are shown below (Table 2), and all obtained results are presented in the Supplementary Materials (Tables S1 and S2).

**Figure 2:**
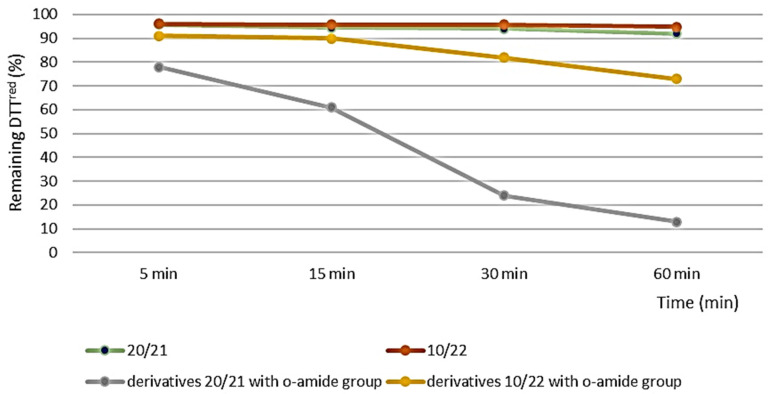
Results of the antioxidant activity measurements of **20**/**21**, **10**/**22** and corresponding Se-derivatives with *o*-amide group.

The best result was observed for *O*-(propyl)-2-((2-oxopropyl)selanyl)benzoate **14**. However, the conversion obtained for all derivatives was less efficient than for ebselen and the previously obtained derivatives with the *o*-amide group. As presented in Figure 2, it can be observed that the presence of an *o*-ester substituent instead of an *o*-amide group in the structure of *β*-carbonyl selenides significantly lowers the ability to reduce H_2_O_2_. In the case of enantiomers **20**/**21** and their counterparts with the o-amide group, these properties are reduced by as much as 9 times.

During our previous research, we also obtained a series of benzisoselenazolones and diselenides *N*-functionalized with long carbon chains terminated with ester groups. The obtained results indicate high reactivity of compounds possessing a Se–N bond along with the ester functionality. This suggests that the isoselenazolone core is essential for the elevated antioxidant potential of the ester-type Se-derivatives [25]. 

**Figure 3:**
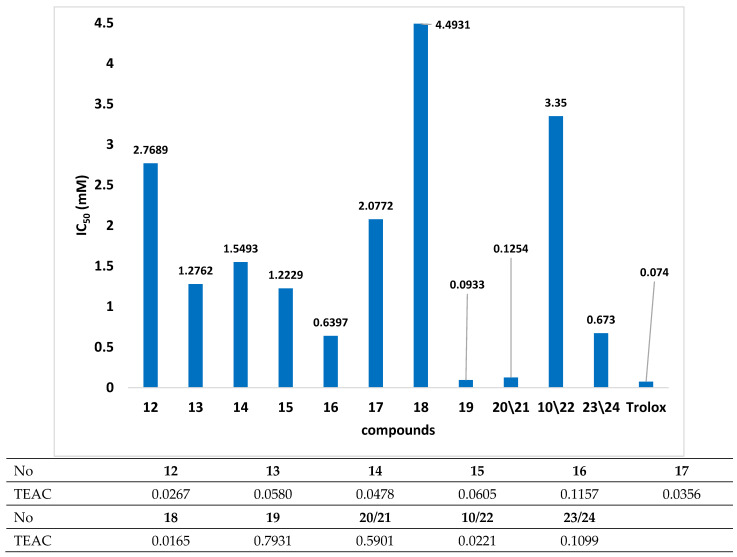
The results of DPPH test for tested compounds where TEAC–Trolox equivalent antioxidant capacity (IC_50_Trolox/IC_50_ compounds) was calculated.

Because organoselenium compounds are well known for their ability to act as antioxidants and/or pro-oxidants, the compounds obtained were tested for their capacities to scavenge free radicals. The results obtained for the DPPH assay are presented in Figure 3 and listed in Table S1 (Supplementary Materials).

In this study, the selected *β*-carbonyl selenides with the *o*-ester group were tested, and the loss of DPPH radical absorbance was detected in the presence of all. As observed, compound **19** exhibited the best free-radical scavenging capabilities. The calculated IC_50_ value was 0.0933 mM at 15 min post-reaction initiation. Given the IC_50_ value obtained for Trolox (0.0744 mM) and the remaining tested compounds, the free-radical scavenging capacity of compound **19** can be considered noticeable. 

All tested organoselenium derivatives contained the same 2-((2-oxopropyl)selenyl)benzoate core but differed in the attached esterification functional substituents. *O*-((1*R*,2*S*,5*R*)-(−)-2-isopropyl-5-methylcyclohexyl)-2-((2-oxopropyl)selanyl)benzoate **19** differed from the other studied derivatives by the presence of a terpene moiety in its structure. It surprised us because monoterpenes without conjugated π bonds, such as menthol, did not have satisfactory free-radical scavenging properties in the DPPH assay [26] and antioxidant activity in the ABTS test [27]. In our previous research, we obtained a new group of chiral benzisoselenazol-3(2*H*)-ones substituted on the nitrogen atom with monoterpene moieties, among others, *p*-menthane [28]. The antioxidant activities of synthesized benzisoselenazolones (such as *N*-menthyl-1,2-benzisoselenazol-3(2*H*)-one) were evaluated based on the Iwaoka test [24]. The pinene derivatives were the most effective, whereas the substrate conversion observed for the menthane derivative after 60 min was only 67%. However, this compound was characterized by the best anticancer activity against MCF-7 cells [28]. The latter, the literature data and the values of IC_50_ obtained in this study suggest that the presence of terpene moiety [26,27] in the structure may allow compounds with interesting antioxidant and anticancer properties to be obtained.

In the case of the remaining tested compounds, the organoselenium derivatives of chiral alcohols (**20**/**21**, **10**/**22** and **23**/**24**) exhibited better free-radical scavenging properties compared to those derived from aliphatic alcohols (**12**–**17**) or benzyl alcohol (**18**). Moreover, the capacity for free-radical inhibition among this group of compounds was the highest for butyl esters and the lowest for products incorporating methylbenzyl in their structure.

The inferior properties of inhibiting free radicals for organoselenium derivatives of aliphatic alcohols may stem from the absence of π bonds in the functional moiety. However, compound **17**, containing a double bond, was not characterized by better free-radical quenching properties. The lowest TEAC value (0.0166) was determined for compound **18**, which may be due to the appearance of an aromatic ring in the structure. A similar situation was observed by Wojtunik et al. [26] for *p*-cymene. It indicates that further research on these compounds should concentrate on elucidating their mechanisms of action as free-radical inhibitors.

**Figure 4:**
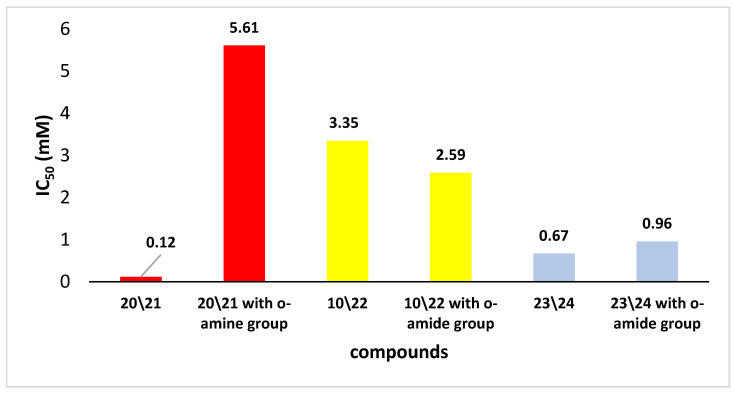
The IC_50_ for the DPPH test of **20**/**21**, **10**/**22** and **23**/**24** and corresponding Se-derivatives with the *o*-amide group.

The literature extensively discusses the relationship between the structures of well-known antioxidants (such as polyphenols) and their antioxidant activity. However, for the discussed compounds, the literature data are very scarce. For this reason, the IC_50_ values for the selected and obtained derivatives were compared with the results for analogous organoselenium compounds described previously [23] (Figure 4).

Given the obtained results, it is difficult to estimate how the replacement of functional groups allowed for organoselenium derivatives with better properties to inhibit the action of free radicals. For example, in the case of compounds **23**/**24**, these properties increased modestly, while for compounds with a s*ec*-butyl group (**20**/**21**), they increased significantly. On the other hand, we did not notice a decrease in the IC_50_ value for derivatives with an *α*-methyl benzyl group **10**/**22**. Generally, one of the compounds with the highest reactivity for derivatives from the *o*-amide group was observed for *N*-(*trans*)-indanyl selenide, for which the IC_50_ value was 0.96 mM. Similar compounds synthesized in this work with an indanyl moiety **23**/**24** but without a hydroxyl group showed an even higher ability to scavenge free radicals.

Finally, the cytotoxic activity of the obtained compounds **10** and **12**–**24** against breast cancer MCF-7 and human promyelocytic leukemia HL-60 cell lines were assessed using the cell viability assay MTT [29]. The highest cytotoxic potential was observed for the methyl *β*-carbonyl selenoesters **12** for the MCF-7 cell line and *sec*-amyl *β*-carbonyl selenoesters **16** for the HL-60 cell line. All results are shown in Table 3.

Previously, we determined the IC_50_ values for selenium derivatives like benzisoselenazolones, diselenides, phenyl selenides and *β*-carbonyl selenides with the *o*-amide group substituted with different achiral and chiral moieties, also in the form of enantiomeric and diastereomeric pairs. Through the analysis of these results, we can see the differentiation of values between individual groups of compounds and between enantiomers and diastereoisomers. As a representative example, a comparison of the results obtained for enantiomers **10**/**22** and corresponding Se-derivatives is presented (Table 4).

Comparing the IC_50_ values obtained for the same *α*-methylbenzyl derivatives, we can notice that for both molecules with an *o*-ester and an *o*-amide group, the best activity was achieved for the *S* configuration for the HL-60 cell line. Additionally, a greater sensitivity of the HL-60 cell line compared to the MCF-7 cell line to the obtained derivatives with an α-methylbenzyl group can be noticed. We can also see an increase in the anticancer properties of *o*-ester selenides compared to phenyl selenides. In general, we can observe that the exchange of the *o*-amide to o-ester moiety enhances the cytotoxic potential of the obtained *β*-carbonyl selenides. 

An important scaffold in our research, which is particularly interesting due to the chirality of compounds and the change in biological activity for individual enantiomers and diastereoisomers, was the hydroxyindanyl moiety. In this work, for *β*-carbonyl selenide with the *o*-ester group, we obtained derivatives with an indanyl substituent without an additional hydroxyl group; therefore, the values can not be compared in a direct way. Looking at the results for the other groups of compounds, we can notice lower sensitivity of cancer cells to compounds **23** and **24,** but it is difficult to determine whether this is the effect of replacing the amide group with an ester group in the *ortho* position or the lack of an additional hydroxyl group.

Looking ahead, our primary goals will involve advancing our understanding of apoptosis and DNA damage pathways, which are fundamental to the cytotoxicity mechanisms. By unravelling the molecular mechanisms governing these pathways, we will aim to elucidate the underlying basis for the anticancer effects of our compounds. Understanding apoptosis and DNA damage pathways is crucial because they regulate cell death and survival, especially in cancer.

## 3. Materials and Methods

### 3.1. General

The 1H, 13C NMR and 77Se NMR spectra were recorded on Bruker Avance III/400 or Bruker Avance III/700 (Karlsruhe, Germany) and 176.1 MHz or 100.6 MHz (Supplementary Materials). The spectra were made in *d*_6_-DMSO solvent (CD_3_OD δ3.31), and chemical shifts were recorded relative to SiMe_4_ or diphenyl diselenide as an external standard. NMR spectra were edited using ACD/NMR Processor Academic Edition. Multiplicities were given as s (singlet), d (doublet), dd (double doublet), t (triplet), dt (double triplet), q (quartet), sextet, septet and m (multiplet). Melting points were measured on the Büchi Tottoli SPM-20 heating unit (Büchi Labortechnik AG, Flawil, Switzerland) and were uncorrected. Vario MACRO CHN analyzer was used for elemental analyses. Optical rotations were measured with a polAAr 3000 polarimeter (Bury Road Industrial Estate, Ramsey, UK). 

For column chromatography, Merck 40-63D 60Å silica gel (Merck, Darmstadt, Germany) was used. Alcohols were commercially available from Merck (Merck, Darmstadt, Germany). Their chemical purities were above 98%, and optical purities were above 97%. The MCF-7 (human breast adenocarcinoma) cell line was obtained from the European Collection of Cell Cultures (ECACC, Nice, France), and the HL-60 cell line (human leukemia) was obtained from the European Collection of Authenticated Cell Cultures (ECACC Nice, France).

### 3.2. General Procedure and Analysis Data

To acetone (2 mL) was added NaHCO_3_ (0.063 g, 0.75 mmol, 1 eq.) (**12**–**15**) or Et_3_N (0.095 g, 0.75 mmol, 1 eg) (**10**, **16**–**24**) with stirring for 30 min; then, the solution of 2-(chloroseleno)-benzoyl chloride (0.190 g, 0.75 mmol, 1 eq.) in acetone (1 mL) was added. After 1 h, alcohol (1 mmol, 1.3 eq.) was added, and the reaction was continued for 15 h at room temperature. The reaction was completed with water (10 mL) and was extracted with DCM. The combined organic layers were dried under magnesium sulfate, and the solvent was evaporated. The product was purified by column chromatography (silica gel, DCM).

*O*-(methyl)-2-((2-oxopropyl)selanyl)benzoate **12**

Yield: 74%; mp 74–75 °C;

^1^H NMR (400 MHz, DMSO) δ = 2.27 (s, 3H), 3.87 (s, 2H), 3.90 (s, 1H), 7.32 (t, *J* = 7.7 Hz, 1H_ar_), 7.52–7.59 (m, 2H_ar_), 7.98 (d, *J* = 7.7 Hz, 1H_ar_) ^13^C NMR (400 MHz, DMSO) δ = 28.92 (CH_3_), 35.44 (CH_2_), 52.88 (CH_2_), 125.75 (CH_ar_), 127.98 (C_ar_), 128.98 (CH_ar_), 131.69 (CH_ar_), 133.65 (CH_ar_), 136.83 (C_ar_), 167.04 (C=O), 204.90 (C=O) ^77^Se NMR (400 MHz, DMSO) δ = 339.57 ppm; IR: 2951, 2913, 1693, 1583, 1460, 1433, 1397, 1357, 1288, 1274, 1252, 1193, 1145, 1101, 1056, 1031, 958, 747, 688 cm^−1^. Elemental Anal. Calcd for C_11_H_12_O_3_Se (271.17): C, 48.72; H, 4.46; Found C, 48.63; H, 4.57.

*O*-(ethyl)-2-((2-oxopropyl)selanyl)benzoate **13**

Yield: 70%; mp 60–61 °C; 

^1^H NMR (400 MHz, DMSO) δ = 1.32 (t, *J* = 7.2 Hz, 3H), 2.25 (s, 3H), 3.87 (s, 2H), 4.99 (q, *J* = 7.2 Hz, 1H), 7.30 (t, *J* = 7.2 Hz, 1H_ar_), 7.47–7.56 (m, 2H_ar_), 7.96 (d, *J* = 7.2 Hz, 1H_ar_) ^13^C NMR (400 MHz, DMSO) δ = 14.59 (CH_3_), 28.93 (CH_3_), 35.46 (CH_2_), 61.69 (CH_2_), 125.74 (CH_ar_), 128.31 (C_ar_), 128.97 (CH_ar_), 131.64 (CH_ar_), 133.57 (CH_ar_), 136.70 (C_ar_), 166.59 (C=O), 204.92 (C=O) ^77^Se NMR (400 MHz, DMSO) δ = 338.99 ppm; IR: 2990, 2976, 2910, 2852, 1688, 1582, 1561, 1457, 1398, 1358, 1304, 1286, 1265, 1248, 1145, 1097, 1053, 1031, 1020, 961, 745, 687 cm^−1^. Elemental Anal. Calcd for C_12_H_14_O_3_Se (285.20): C, 53.98; H, 4.65; Found C, 53.81; H, 4.71.

*O*-(propyl)-2-((2-oxopropyl)selanyl)benzoate **14**

Yield: 57%; 

^1^H NMR (700 MHz, DMSO) δ = 0.98 (t, *J* = 7.0 Hz, 3H), 1.74 (sextet, *J* = 6.3 Hz, 2H), 2.27 (s, 3H), 3.90 (s, 2H), 4.25 (t, *J* = 7.0 Hz, 2H), 7.33 (t, *J* = 7.0 Hz, 1H_ar_), 7.50–7.58 (m, 2H_ar_), 7.99 (d, *J* = 7.7 Hz, 1H_ar_) ^13^C NMR (400 MHz, DMSO) δ = 10.86 (CH_3_), 22.04 (CH_2_), 28.93 (CH_3_), 35.45 (CH_2_), 67.01 (CH_2_), 125.77 (CH_ar_), 128.26 (C_ar_), 128.98 (CH_ar_), 131.62 (CH_ar_), 133.60 (CH_ar_), 136.74 (C_ar_), 166.64 (C=O), 204.93 (C=O) ^77^Se NMR (700 MHz, DMSO) δ = 338.76 ppm; IR: 2969, 2878, 1696, 1584, 1535, 1459, 1354, 1305, 1272, 1227, 1143, 1100, 1056, 1032, 739 cm^−1^. Elemental Anal. Calcd for C_13_H_16_O_3_Se (299.22): C, 52.18; H, 5.39; Found C, 52.31; H, 5.55.

*O*-(2-propyl)-2-((2-oxopropyl)selanyl)benzoate **15**

Yield: 65%;

^1^H NMR (400 MHz, DMSO) δ = 1.31 (d, *J* = 6.4 Hz, 6H), 2.25 (s, 3H), 3.86 (s, 2H), 5.13 (septet, *J* = 6.0 Hz, 1H), 7.30 (t, *J* = 6.4 Hz, 1H_ar_), 7.46–7.55 (m, 2H_ar_), 7.93 (d, *J* = 6.8 Hz, 1H_ar_) ^13^C NMR (400 MHz, DMSO) δ = 22.11 (2×CH_3_), 28.94 (CH_3_), 35.49 (CH_2_), 69.36 (CH), 125.72 (CH_ar_), 128.73 (C_ar_), 128.97 (CH_ar_), 131.57 (CH_ar_), 133.49 (CH_ar_), 136.54 (C_ar_), 166.13 (C=O), 204.92 (C=O) ^77^Se NMR (400 MHz, DMSO) δ = 337.89 ppm; IR: 2980, 2937, 1693, 1584, 1455, 1354, 1273, 1254, 1226, 1146, 1098, 1054, 1032, 738 cm^−1^. Elemental Anal. Calcd for C_13_H_16_O_3_Se (299.22): C, 52.18; H, 5.39; Found C, 52.42; H, 5.48.

*O*-(2-pentyl)-2-((2-oxopropyl)selanyl)benzoate **16**

Yield: 39%;

^1^H NMR (700 MHz, DMSO) δ = 0.91 (t, *J* = 7.0 Hz, 3H), 1.30 (d, *J* = 7.0 Hz, 3H), 1.33–1.45 (m, 2H), 1.56–1.64 (m, 1H), 1.65–1.73 (m, 1H), 2.27 (s, 3H), 3.89 (s, 2H), 5.08 (sextet, *J* = 7.0 Hz, 1H), 7.32 (t, *J* = 7.0 Hz, 1H_ar_), 7.50–7.58 (m, 2H_ar_), 7.96 (d, *J* = 7.7 Hz, 1H_ar_) ^13^C NMR (700 MHz, DMSO) δ = 14.22 (CH_3_), 18.62 (CH_2_), 20.28 (CH_3_), 28.94 (CH_3_), 35.50 (CH_2_), 37.92 (CH_2_), 72.26 (CH), 125.76 (CH_ar_), 128.66 (C_ar_), 128.99 (CH_ar_), 131.51 (CH_ar_), 133.51 (CH_ar_), 136.61 (C_ar_), 166.23 (C=O), 204.94 (C=O) 77Se NMR (700 MHz, DMSO) δ = 339.07 ppm; IR: 2958, 2931, 2872, 1693, 1583, 1458, 1356, 1303, 1275, 1254, 1226, 1183, 1145, 1099, 1054, 1031, 738, 688 cm^−1^. Elemental Anal. Calcd for C_15_H_20_O_3_Se (327.28): C, 55.05; H, 6.16; Found C, 55.23; H, 6.24.

*O*-(3-methylbut-2-en-1-yl)-2-((2-oxopropyl)selanyl)benzoate **17**

Yield: 34%;

^1^H NMR (700 MHz, DMSO) δ = 1.75 (d, *J* = 9.1 Hz, 6H), 2.27 (s, 3H), 3.89 (s, 2H), 4.80 (d, *J* = 7.0 Hz, 2H), 5.44 (t, *J* = 7.0 Hz, 1H), 7.32 (t, *J* = 7.0 Hz, 1H_ar_), 7.49–7.58 (m, 2H_ar_), 7.96 (d, *J* = 7.7 Hz, 1H_ar_) ^13^C NMR (400 MHz, DMSO) δ = 18.42 (CH_3_), 25.91 (CH_3_), 28.93 (CH_3_), 35.46 (CH_2_), 62.27 (CH_2_), 118.95 (CH), 125.74 (CH_ar_), 128.23 (C_ar_), 128.95 (CH_ar_), 131.67 (CH_ar_), 133.59 (CH_ar_), 136.77 (C_ar_), 139.59 (C_ar_), 166.52 (C=O), 204.92 (C=O) ^77^Se NMR (400 MHz, DMSO) δ = 339.21 ppm; IR: 2971, 2938, 1690, 1585, 1439, 1358, 1270, 1228, 1146, 1095, 1053, 1031, 942, 738, 687 cm^−1^. Elemental Anal. Calcd for C_15_H_18_O_3_Se (325.26): C, 55.39; H, 5.56; Found C, 55.61; H, 5.72.

*O*-(benzyl)-2-((2-oxopropyl)selanyl)benzoate **18**

Yield: 38%;

^1^H NMR (700 MHz, DMSO) δ = 2.27 (s, 3H), 3.91 (s, 2H), 5.37 (s, 2H), 7.30–7.35 (m, 2H_ar_), 7.35–7.39 (m, 1H_ar_) 7.42 (t, *J* = 7.7 Hz, 2H_ar_), 7.47–7.50 (m, 2H_ar_), 7.52–7.58 (m, 2H_ar_), 8.02 (d, *J* = 7.7 Hz, 1H_ar_) ^13^C NMR (400 MHz, DMSO) δ = 28.94 (CH_3_), 35.46 (CH_2_), 67.07 (CH), 125.83 (CH_ar_), 127.88 (C_ar_), 128.58 (2×CH_ar_), 128.71 (CH_ar_), 129.03 (2×CH_ar_), 129.45 (CH_ar_), 131.79 (CH_ar_), 133.79 (CH_ar_), 136.32 (C_ar_), 137.01 (C_ar_), 166.38 (C=O), 204.96 (C=O) ^77^Se NMR (400 MHz, DMSO) δ = 339.53 ppm; IR: 3030, 2924, 2853, 1696, 1584, 1631, 1455, 1355, 1303, 1270, 1251, 1227, 1141, 1096, 1053, 1031, 967, 738, 696 cm^−1^. Elemental Anal. Calcd for C_17_H_16_O_3_Se (347.27): C, 58.80; H, 4.64; Found C, 59.04; H, 4.71.

*O*-((1*R,*2*S,*5*R*)-(−)-2-isopropyl-5-methylcyclohexyl)-2-((2-oxopropyl)selanyl)benzoate **19**

Yield: 18%; ${\left[\propto \right]}_{D}^{20}$ = −29 (c = 0.20, CHCl_3_);

^1^H NMR (700 MHz, DMSO) δ = 0.76 (d, *J* = 7 Hz, 3H), 0.89 (d, *J* = 7 Hz, 3H), 0.91 (d, *J* = 7 Hz, 3H), 1.08–1.18 (m, 2H), 1.22–1.29 (m, 1H), 1.48–1.60 (m, 2H), 1.84–1.94 (m, 2H), 1.97–2.05 (m, 1H), 2.22–2.28 (m, 1H), 2.27 (s, 3H), 3.89 (s, 2H), 4.86 (td, J_1_ = 4.9 Hz, J_2_ = 11.2 Hz, 1H), 7.33 (t, *J* = 7.0 Hz, 1H_ar_), 7.47–7.56 (m, 2H_ar_), 7.95 (d, *J* = 7.7 Hz, 1H_ar_) ^13^C NMR (400 MHz, DMSO) δ = 16.86 (CH_3_), 20.94 (CH_3_), 22.33 (CH_3_), 23.62 (CH_2_), 26.64 (CH), 28.95 (CH_3_), 31.37 (CH), 34.15 (CH_2_), 35.54 (CH_2_), 40.98 (CH_2_), 47.05 (CH), 75.30 (CH), 125.81 (CH_ar_), 128.49 (C_ar_), 129.04 (CH_ar_), 131.46 (CH_ar_), 133.56 (CH_ar_), 136.76 (C_ar_), 166.08 (C=O), 204.93 (C=O) ^77^Se NMR (400 MHz, DMSO) δ = 338.30 ppm; IR: 2953, 2923, 2868, 1692, 1585, 1460, 1358, 1272, 1254, 1228, 1142, 1098, 1052, 1031, 958, 738 cm^−1^. Elemental Anal. Calcd for C_20_H_28_O_3_Se (395.39): C, 60.75; H, 7.14; Found C, 60.89; H, 7.22.

*O*-((*S*)-(+)-*sec*-butyl)-2-((2-oxopropyl)selanyl)benzoate **20**

Yield: 16%; ${\left[\propto \right]}_{D}^{20}$ = 50 (c = 0.40, CHCl_3_);

^1^H NMR (700 MHz, DMSO) δ = 0.90 (t, *J* = 7.0 Hz, 3H), 1.28 (d, *J* = 11.2 Hz, 3H), 1.58–1.72 (m, 2H), 2.25 (s, 3H), 3.85 (s, 2H), 4.99 (sextet, *J* = 7.0 Hz 1H), 7.26–7.32 (m, 1H_ar_), 7.44–7.57 (m, 2H_ar_), 7.91–7.99 (dd, J_1_ = 2.1 Hz, J_2_ = 9.8 Hz, 1H_ar_) ^13^C NMR (700 MHz, DMSO) δ = 10.00 (CH_3_), 19.75 (CH_3_), 28.73 (CH_2_), 28.95 (CH_3_), 35.48 (CH_2_), 73.71 (CH), 125.77 (CH_ar_), 128.66 (C_ar_), 128.98 (CH_ar_), 131.52 (CH_ar_), 133.52 (CH_ar_), 136.59 (C_ar_), 167.27 (C=O), 204.99 (C=O) ^77^Se NMR (700 MHz, DMSO) δ = 338.89 ppm; IR: 2969, 2924, 1693, 1584, 1457, 1434, 1355, 1304, 1273, 1254, 1226, 1144, 1127, 1099, 1053, 1029, 965, 739, 688 cm^−1^. Elemental Anal. Calcd for C_14_H_18_O_3_Se (313.25): C, 53.68; H, 5.79; Found C, 53.97; H, 5.86.

*O*-((*R*)-(−)-*sec*-butyl)-2-((2-oxopropyl)selanyl)benzoate **21**

Yield: 22%; ${\left[\propto \right]}_{D}^{20}$ = −46 (c = 0.35, CHCl_3_);

^1^H NMR (700 MHz, DMSO) δ = 0.92 (t, *J* = 7.0 Hz, 3H), 1.30 (d, *J* = 7.0 Hz, 3H), 1.59–1.72 (m, 2H), 2.27 (s, 3H), 3.88 (s, 2H), 5.01 (sextet, *J* = 7.0 Hz, 1H), 7.31–7.36 (m, 1H_ar_), 7.47–7.56 (m, 2H_ar_), 7.91–7.99 (dd, J_1_ = 1.4 Hz, J_2_ = 7 Hz, 1H_ar_) ^13^C NMR (700 MHz, DMSO) δ = 10.00 (CH_3_), 19.75 (CH_3_), 28.74 (CH_2_), 28.95 (CH_3_), 35.49 (CH_2_), 73.71 (CH), 125.77 (CH_ar_), 128.67 (C_ar_), 128.99 (CH_ar_), 131.52 (CH_ar_), 133.52 (CH_ar_), 136.59 (C_ar_), 167.27 (C=O), 204.98 (C=O) ^77^Se NMR (700 MHz, DMSO) δ = 338.91 ppm; IR: 2969, 2924, 1693, 1584, 1457, 1355, 1304, 1273, 1254, 1226, 1144, 1127, 1099, 1052, 1028, 966, 739, 688 cm^−1^. Elemental Anal. Calcd for C_14_H_18_O_3_Se (313.25): C, 53.68; H, 5.79; Found C, 53.54; H, 5.71.

*O*-((*R*)-(+)-α-methylbenzyl)-2-((2-oxopropyl)selanyl)benzoate **10**

Yield: 54%; ${\left[\propto \right]}_{D}^{20}$ = 58 (c = 0.24, CHCl_3_);

^1^H NMR (400 MHz, DMSO) δ = 1.60 (d, *J* = 6.4 Hz, 3H), 2.23 (s, 3H), 3.86 (s, 2H), 6.04 (q, *J* = 6.8 Hz, 1H), 7.27–7.35 (m, 2H_ar_), 7.38 (t, *J* = 6.8 Hz, 2H_ar_), 7.42–7.49 (m, 2H_ar_), 7.51–7.56 (m, 2H_ar_), 8.07 (d, *J* = 7.2 Hz, 1H_ar_) ^13^C NMR (400 MHz, DMSO) δ = 22.78 (CH_3_), 28.94 (CH_3_), 35.47 (CH_2_), 73.71 (CH), 125.82 (CH_ar_), 126.39 (2×CH_ar_), 128.11 (C_ar_), 128.38 (CH_ar_), 128.98 (CH_ar_), 129.02 (2×CH_ar_), 131.80 (CH_ar_), 133.75 (CH_ar_), 136.99 (C_ar_), 141.99 (C_ar_), 165.77 (C=O), 204.91 (C=O) ^77^Se NMR (400 MHz, DMSO) δ = 339.53 ppm; IR: 2980, 2929, 1695, 1584, 1456, 1355, 1302, 1270, 1252, 1144, 1099, 1053, 1029, 993, 760, 698 cm^−1^. Elemental Anal. Calcd for C_14_H_18_O_3_Se (361.29): C, 59.84; H, 5.02; Found C, 59.98; H, 4.95.

*O*-((*S*)-(−)-α-methylbenzyl)-2-((2-oxopropyl)selanyl)benzoate **22**

Yield: 21%; ${\left[\propto \right]}_{D}^{20}$ = −59 (c = 0.32, CHCl_3_);

^1^H NMR (700 MHz, DMSO) δ = 1.62 (d, *J* = 5.6 Hz, 3H), 2.25 (s, 3H), 3.88 (s, 2H), 6.05 (q, *J* = 6.3 Hz, 1H), 7.29–7.36 (m, 2H_ar_), 7.40 (t, *J* = 6.3 Hz, 2H_ar_), 7.46–7.52 (m, 2H_ar_), 7.53–7.58 (m, 2H_ar_), 8.09 (d, *J* = 6.3 Hz, 1H_ar_) ^13^C NMR (400 MHz, DMSO) δ = 22.77 (CH_3_), 28.94 (CH_3_), 35.47 (CH_2_), 73.71 (CH), 125.82 (CH_ar_), 126.38 (2×CH_ar_), 128.13 (C_ar_), 128.32 (CH_ar_), 129.00 (CH_ar_), 129.01 (2×CH_ar_), 131.79 (CH_ar_), 133.74 (CH_ar_), 136.97 (C_ar_), 141.98 (C_ar_), 165.77 (C=O), 204.89 (C=O) ^77^Se NMR (400 MHz, DMSO) δ = 339.57 ppm; IR: 2979, 2929, 1694, 1583, 1456, 1355, 1301, 1270, 1252, 1143, 1099, 1052, 1028, 993, 760, 698 cm^−1^. Elemental Anal. Calcd for C_14_H_18_O_3_Se (361.29): C, 59.84; H, 5.02; Found C, 60.02; H, 5.12.

*O*-((*R*)-(−)-2,3-dihydro-(1*H)*-inden-1-yl)-2-((2-oxopropyl)selanyl)benzoate **23**

Yield: 28%; ${\left[\propto \right]}_{D}^{20}$ = −27 (c = 0.31, CHCl_3_);

^1^H NMR (700 MHz, DMSO) δ = 2.14–2.23 (m, 1H), 2.27 (s, 3H), 2.55–2.61 (m, 1H), 2.88–2.97 (m, 1H), 3.09–3.17 (m, 1H), 3.91 (s, 2H), 6.36 (q, *J* = 2.1 Hz, 1H), 7.22–7.30 (m, 2H_ar_), 7.33–7.40 (m, 2H_ar_), 7.48 (d, *J* = 1.4 Hz, 1H_ar_), 7.51–7.57 (m, 2H_ar_), 7.91 (d, *J* = 7.7 Hz, 1H_ar_) ^13^C NMR (700 MHz, DMSO) δ = 28.97 (CH_3_), 30.24 (CH_2_), 32.37 (CH_2_), 35.55 (CH_2_), 79.71 (CH), 125.37 (CH_ar_), 125.77 (CH_ar_), 125.92 (CH_ar_), 127.17 (CH_ar_), 128.37 (C_ar_), 129.03 (CH_ar_), 129.58 (CH_ar_), 131.76 (CH_ar_), 133.64 (CH_ar_), 136.73 (C_ar_), 141.04 (C_ar_), 144.86 (C_ar_), 166.56 (C=O), 204.91 (C=O) ^77^Se NMR (400 MHz, DMSO) δ = 337.85 ppm; IR: 2970, 2927, 2852, 1692, 1584, 1460, 1355, 1271, 1251, 1227, 1141, 1099, 1053, 1031, 738 cm^−1^. Elemental Anal. Calcd for C_19_H_18_O_3_Se (373.30): C, 61.13; H, 4.86; Found C, 61.01; H, 4.97.

*O*-((*S*)-(+)-2,3-dihydro-(1*H*)-inden-1-yl)-2-((2-oxopropyl)selanyl)benzoate **24**

Yield: 25%; ${\left[\propto \right]}_{D}^{20}$ = 24 (c = 0.37, CHCl_3_);

^1^H NMR (700 MHz, DMSO) δ = 2.14–2.22 (m, 1H), 2.27 (s, 3H), 2.53–2.60 (m, 1H), 2.90–2.97 (m, 1H), 3.08–3.18 (m, 1H), 3.91 (s, 2H), 6.38 (q, *J* = 3.5 Hz, 1H), 7.21–7.30 (m, 2H_ar_), 7.30–7.40 (m, 2H_ar_), 7.48 (d, *J* = 7.7 Hz, 1H_ar_), 7.52–7.58 (m, 2H_ar_), 7.91 (d, *J* = 7.7 Hz, 1H_ar_) ^13^C NMR (400 MHz, DMSO) δ = 28.97 (CH_3_), 30.24 (CH_2_), 32.36 (CH_2_), 35.54 (CH_2_), 79.70 (CH), 125.36 (CH_ar_), 125.77 (CH_ar_), 125.92 (CH_ar_), 127.17 (CH_ar_), 128.36 (C_ar_), 129.02 (CH_ar_), 129.58 (CH_ar_), 131.76 (CH_ar_), 133.64 (CH_ar_), 136.74 (C_ar_), 141.03 (C_ar_), 144.86 (C_ar_), 166.55 (C=O), 204.90 (C=O) ^77^Se NMR (400 MHz, DMSO) δ = 337.85 ppm; IR: 2970, 2925, 2851, 1694, 1584, 1460, 1354, 1271, 1251, 1227, 1142, 1099, 1053, 1032, 740 cm^−1^. Elemental Anal. Calcd for C_19_H_18_O_3_Se (373.30): C, 61.13; H, 4.86; Found C, 61.26; H, 4.93.

### 3.3. Antioxidant Activity Evaluation 

#### 3.3.1. DTT Activity Assay

DDT activity assay for compounds **10** and **11**–**24** was performed according to the Iwaoka procedure [24].

#### 3.3.2. The 2,2-di(4-tert-Octyl phenyl)-1-picrylhydrazyl) (DPPH) Test

The assay for the neutralization of radicals was executed following the methodology delineated in our previous work [23]. The 50% decline in absorbance of the DPPH solution was derived from the curve with the tested compound concentration (mM) plotted against the absorbance. The obtained results are presented as the inhibitory concentration (IC_50_) of the tested compounds. The absorbance value was measured after 15 min post-initiation. In addition to the compounds that were obtained, the antioxidant activity of Trolox was assessed for comparison purposes. The efficacy of the tested compounds in scavenging DPPH radicals was quantified and expressed as Trolox equivalent antioxidant capacity (TEAC). All details regarding the procedure are included in the Supplementary Materials.

### 3.4. MTT Viability Assay 

The MTT (3-(4,5-didiazol-2-yl)-2,5 diphenyl tetrazolium bromide) assay, which measures the activity of methylcellular dehydrogenases, was based on the method of Mosmann [29]. The MTT assay was executed following the methodology delineated in our previous work [23].

## 4. Conclusions

We developed an efficient method for synthesizing a new group of organoselenium compounds, *β*-carbonyl phenyl selenides, possessing an ester group in the ortho position. The key step in the synthesis was using triethylamine during the acylation of selenide. The first derivatives with alkyl achiral and chiral scaffolds were obtained. The obtained derivatives were tested for antioxidant and cytotoxic activity. The antioxidant activity was tested in two ways: the reduction in peroxide to water (Iwaoka test) and the quenching of free radicals (DPPH test). As a result of these studies, we observed that the exchange of the amide group to an ester group significantly lowers the H_2_O_2_ reduction properties. However, it was observed that the obtained ester derivatives are better free-radical scavengers. The best results were obtained for the compound O-((1*R*,2*S*,5*R*)-(−)-2-isopropyl-5-methylcyclohexyl)-2-((2-oxopropyl)selanyl)benzoate, for which the IC_50_ value was close to the value for Trolox. Very good results of the DPPH test were also obtained for the *sec*-butyl and indanyl derivatives. In the case of cytotoxic activity, which was tested on two cell lines, MCF-7 (breast cancer) and HL-60 (promyelocytic leukemia), we did not notice a relevant improvement in these properties.

## Figures and Tables

**Table 1:** Optimization of reaction conditions.

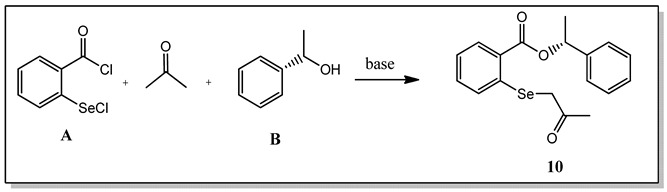				
Entry	Solvent (Solubilization of A)	Base	Conditions and Order of Addition	Yield (%)
1	Acetone	NaHCO_3_	1. (acetone + NaHCO_3_, rt, 30 min) + **A**, rt, 1 h 2. **B**, rt, 15 h	16
2	Acetone	Et_3_N	1. (acetone + Et_3_N, rt, 30 min) + **A**, rt, 1 h 2. **B**, rt, 15 h	54

**Table 2:** Results of the antioxidant activity measurement.

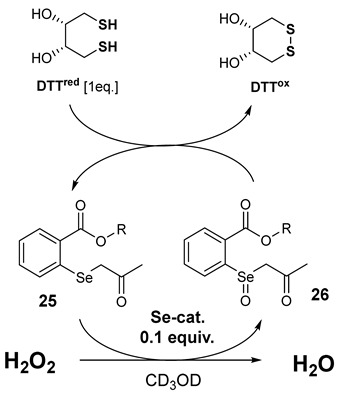				
	Remaining DTT^red^ (%)			
Catalyst (0.1 Equiv.)	5 min	15 min	30 min	60 min
**12**	95.3 ± 0.2	95.2 ± 0.3	94.8 ± 0.4	94.4 ± 0.4
**13**	96.7 ± 0.1	96.5 ± 0.2	95.0 ± 0.1	94.9 ± 0.4
**14**	92.8 ± 0.1	88.5 ± 0.3	82.0 ± 1.9	75.0 ± 1.1
**15**	93.6 ± 0.2	93.0 ± 0.1	92.9 ± 0.2	92.6 ± 0.2
**16**	95.1 ± 0.1	94.9 ± 0.1	94.7 ± 0.1	94.4 ± 0.2
**17**	90.8 ± 0.2	90.6 ± 0.3	90.3 ± 0.3	89.2 ± 0.2
**18**	93.5 ± 0.1	91.5 ± 0.2	89.2 ± 0.3	85.8 ± 0.7
**19**	95.1 ± 0.1	94.9 ± 0.1	94.6 ± 0.1	94.3 ± 0.2
**20/21**	95.8 ± 0.1	94.4 ± 0.2	94.0 ± 1.5	92.1 ± 2.7
**10/22**	96.1 ± 0.1	95.7 ± 0.1	95.7 ± 0.3	94.9 ± 0.4
**23/24**	94.4 ± 0.9	93.9 ± 0.8	93.6 ± 0.7	92.8 ± 0.4
**Ebselen**	75	64	58	52

**Table 3:** The antiproliferative activity of compounds **10** and **12**–**24**.

Compound	IC_50_ (µM) ± SEM	
	MCF-7	HL-60
**12**	51.1 ± 0.6	97.2 ± 0.5
**13**	129.5 ± 1.2	147.0 ± 5.0
**14**	166.0 ± 3.0	97.2 ± 0.1
**15**	273.0 ± 3.0	105.0 ± 1.0
**16**	121.0 ± 0.4	84.0 ± 2.0
**17**	194.0 ± 5.0	103.0 ± 2.0
**18**	133.0 ± 2.0	110.0 ± 5.0
**19**	121.0 ± 10.0	243.0 ± 8.0
**20**	313.0 ± 10.0	147.5 ± 2.0
**21**	243.0 ± 10.0	89.4 ± 0.9
**10**	176.0 ± 3.0	126.0 ± 2.5
**22**	107.0 ± 0.8	94.2 ± 3.0
**23**	123.0 ± 0.4	94.4 ± 3.0
**24**	136.0 ± 3.0	94.2 ± 0.7
**Oxaliplatin**	35 [30,31]	0.8 [32]

**Table 4:** The antiproliferative activity of α-methylbenzyl Se-derivatives.

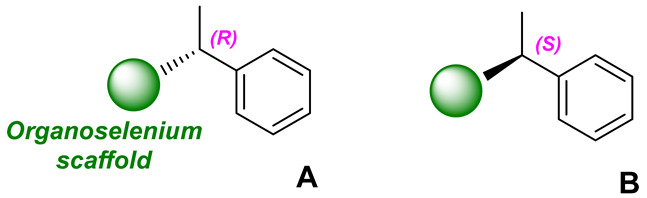				
	IC_50_ (µM) ± SEM			
	A		B	
Se-derivative	MCF-7	HL-60	MCF-7	HL-60
*β*-carbonyl selenide with *o*-ester group	176.0 ± 3.0	126.0 ± 2.5	107.0 ± 0.8	94.2 ± 3.0
*β*-carbonyl selenide with *o*-amide group	235.0 ± 1.0	303.0 ± 3.0	237.0 ± 11.0	23.5 ± 1.4
Phenyl selenide	>150	>150	>150	>150
Benzisoselenazolone	32.8 ± 2.8	16.1 ± 0.0	38.8 ± 0.8	16.8 ± 0.4
Diselenide	>100	>100	>100	>100

## Data Availability

The original contributions presented in the study are included in the article/Supplementary Material, further inquiries can be directed to the corresponding author/s.

## Supplementary Materials

The following supporting information can be downloaded at: https://www.mdpi.com/article/10.3390/molecules29122866/s1, Figure S1: (**a**) 1H NMR, (**b**) 13C NMR, and (**c**) 77Se NMR spectra of *O*-(methyl)- 2-((2-oxopropyl)selanyl)benzoate **12**; Figure S2: (**a**) 1H NMR, (**b**) 13C NMR, and (**c**) 77Se NMR spectra of *O*-(ethyl)- 2-((2-oxopropyl)selanyl)benzoate **13**; Figure S3: (**a**) 1H NMR, (**b**) 13C NMR, and (**c**) 77Se NMR spectra of *O*-(propyl)- 2-((2-oxopropyl)selanyl)benzoate **14**; Figure S4: (**a**) 1H NMR, (**b**) 13C NMR, and (**c**) 77Se NMR spectra of *O*-(2-propyl)- 2-((2-oxopropyl)selanyl)benzoate **15**; Figure S5: (**a**) 1H NMR, (**b**) 13C NMR, and (**c**) 77Se NMR spectra of *O*-(2-pentyl)- 2-((2-oxopropyl)selanyl)benzoate **16**; Figure S6: (**a**) 1H NMR, (**b**) 13C NMR, and (**c**) 77Se NMR spectra of *O*-(3-methylbut-2-en-1-yl)- 2-((2-oxopropyl)selanyl)benzoate **17**; Figure S7: (**a**) 1H NMR, (**b**) 13C NMR, and (**c**) 77Se NMR spectra of *O*-(benzyl)- 2-((2-oxopropyl)selanyl)benzoate **18**; Figure S8; (**a**) 1H NMR, (**b**) 13C NMR, and (**c**) 77Se NMR spectra of *O*-((1*R,*2*S,*5*R*)-(-)-2-isopropyl-5-methylcyclohexyl)-2-((2-oxopropyl)selanyl)benzoate **19**; Figure S9: (**a**) 1H NMR, (**b**) 13C NMR, and (**c**) 77Se NMR spectra of *O*-((*S*)-(+)-*sec*-butyl)- 2-((2-oxopropyl)selanyl)benzoate **20**; Figure S10: (**a**) 1H NMR, (**b**) 13C NMR, and (**c**) 77Se NMR spectra of *O*-((*R*)-(-)-*sec*-butyl)- 2-((2-oxopropyl)selanyl)benzoate **21**; Figure S11: (**a**) 1H NMR, (**b**) 13C NMR, and (**c**) 77Se NMR spectra of *O*-((*R*)-(+)-α-methylbenzyl)- 2-((2-oxopropyl)selanyl)benzoate **10**; Figure S12: (**a**) 1H NMR, (**b**) 13C NMR, and (**c**) 77Se NMR spectra of *O*-((*S*)-(-)-α-methylbenzyl)- 2-((2-oxopropyl)selanyl)benzoate **22**; Figure S13: (**a**) 1H NMR, (**b**) 13C NMR, and (**c**) 77Se NMR spectra of *O*-((*R*)-(-)-2,3-dihydro-(1*H)*-inden-1-yl)-2-((2-oxopropyl)selanyl)benzoate **23**; Figure S14: (**a**) 1H NMR, (**b**) 13C NMR, and (**c**) 77Se NMR spectra of *O*-((*S*)-(+)-2,3-dihydro-(1*H)*-inden-1-yl)-2-((2-oxopropyl)selanyl)benzoate **24**; Table S1: Results of antioxidant activity measurement of integration from 1H NMR spectra after reaction time 5 min and 15 min for all compounds; Table S2: Results of antioxidant activity measurement of integration from 1H NMR spectra after reaction time 30 min and 60 min for all compounds; Table S3: The results of DPPH Radical Scavenging Assay.
