# Supplementary material for: Synthesis of a New Class of β-Carbonyl Selenides Functionalized with Ester Groups with Antioxidant and Anticancer Properties—Part II
Source: Molecules. 2024 Jun 16;29(12):2866. doi: 10.3390/molecules29122866 (PMC11206731; doi:10.3390/molecules29122866)
Supplement: Supplementary file 1 [file molecules-29-02866-s001.zip › molecules-3020818-supplementary.pdf]

Supporting Information

Synthesis of a new class of *β*-carbonyl selenides functionalized with ester groups with antioxidant and anticancer properties—Part II

Anna Laskowska [1], Agata J. Pacuła-Miszewska [1], Magdalena Obieziurska-Fabisiak [1], Aneta Jastrzębska [2], Angelika Długosz-Pokorska [3], Katarzyna Gach-Janczak [3] and Jacek Ścianowski [1,*]

[1] Department of Organic Chemistry, Faculty of Chemistry, Nicolaus Copernicus University, 7 Gagarin Street, 87-100 Torun, Poland; annlas@doktorant.umk.pl (A.L.); pacula@umk.pl (A.J.P.-M.); magdao@umk.pl (M.O.-F.)
[2] Department of Analytical Chemistry and Applied Spectroscopy, Faculty of Chemistry, Nicolaus Copernicus University in Torun, 7 Gagarin Street, 87-100 Torun, Poland; aj@umk.pl
[3] Department of Biomolecular Chemistry, Faculty of Medicine, Medical University of Lodz, 6/8 Mazowiecka Street, 92-215 Lodz, Poland; angelika.dlugosz@umed.lodz.pl (A.D.-P.); katarzyna.gach@umed.lodz.pl (K.G.-J.)
* Correspondence: jsch@umk.pl

Table of content

1. 1H, 13C and 77Se NMR spectra of selenides **10**, **12**-**24**, pages 2-22

2. Antioxidant activity measurement, pages 23-24

3. DPPH Radical Scavenging Assay, page 24

1. NMR spectra of 2-((2-oxopropyl)selanyl) benzoate 10,12-24

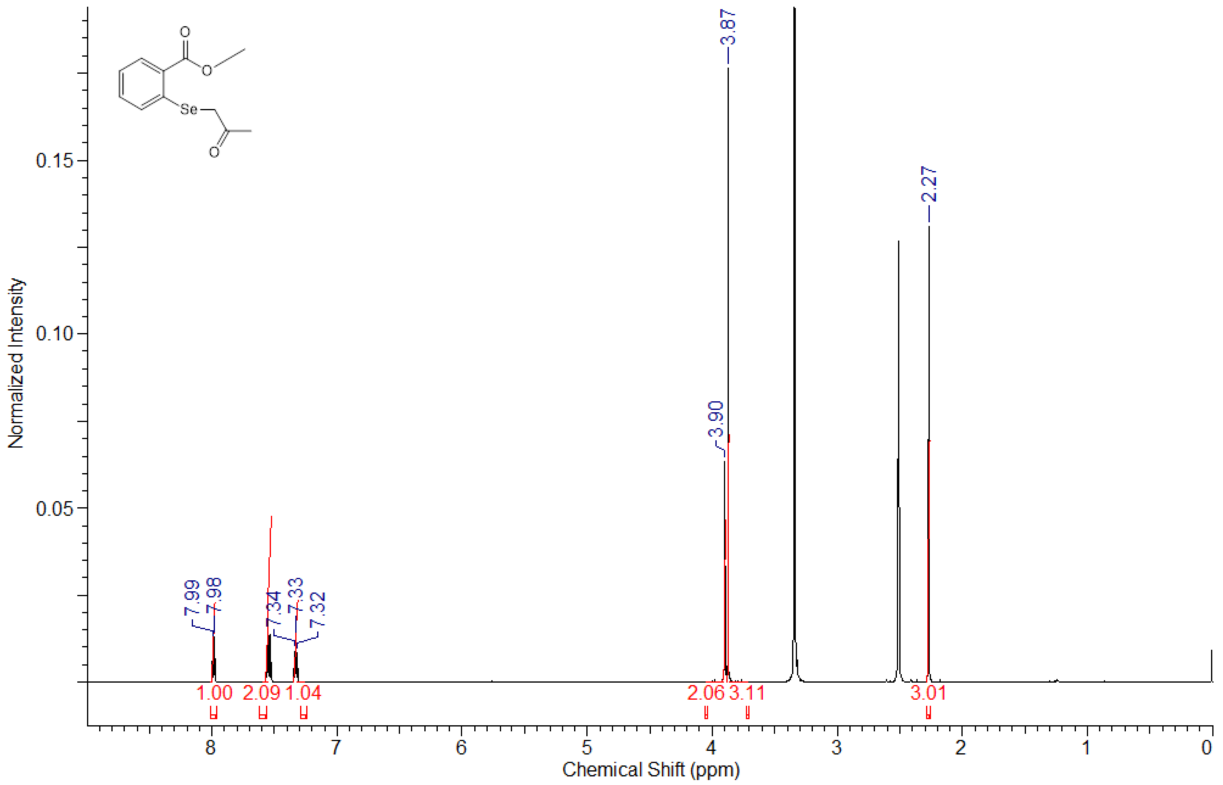

(a)

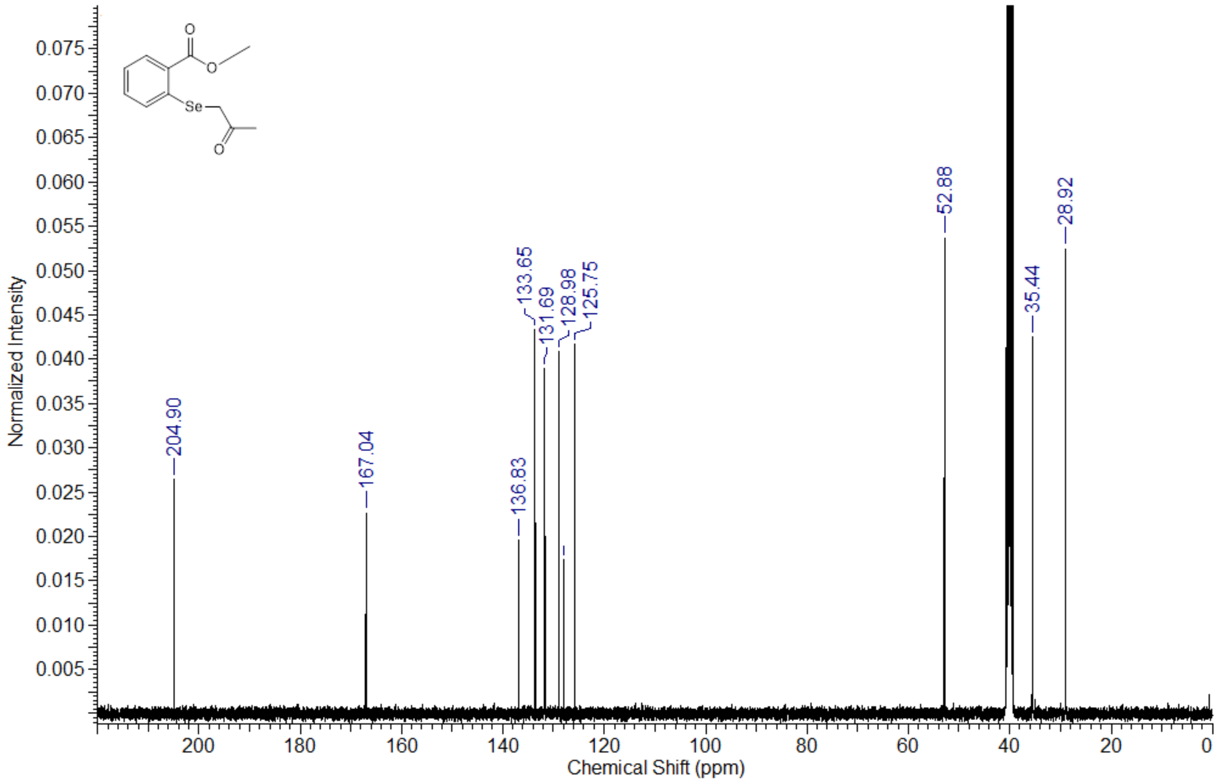

(b)

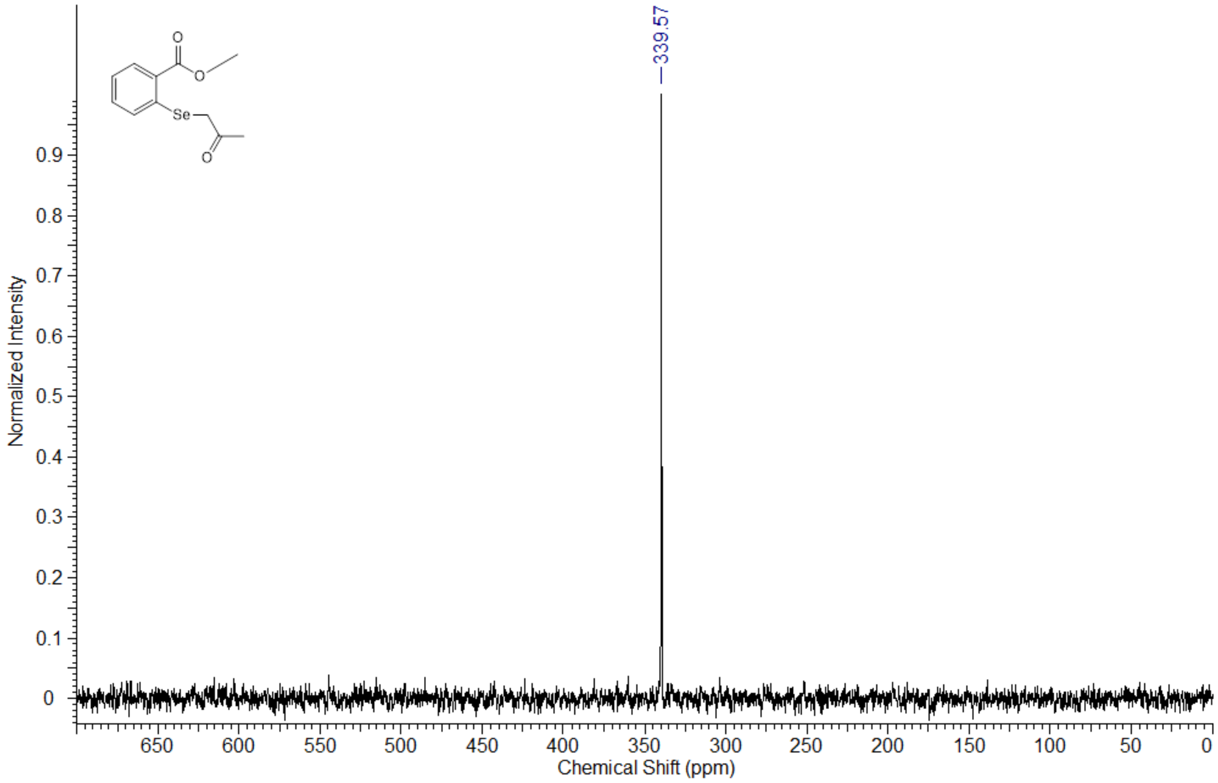

(c)

Figure S1. (**a**) 1H NMR, (**b**) 13C NMR, and (**c**) 77Se NMR spectra of *O*-(methyl)-2-((2-oxopropyl)selanyl)benzoate **12**

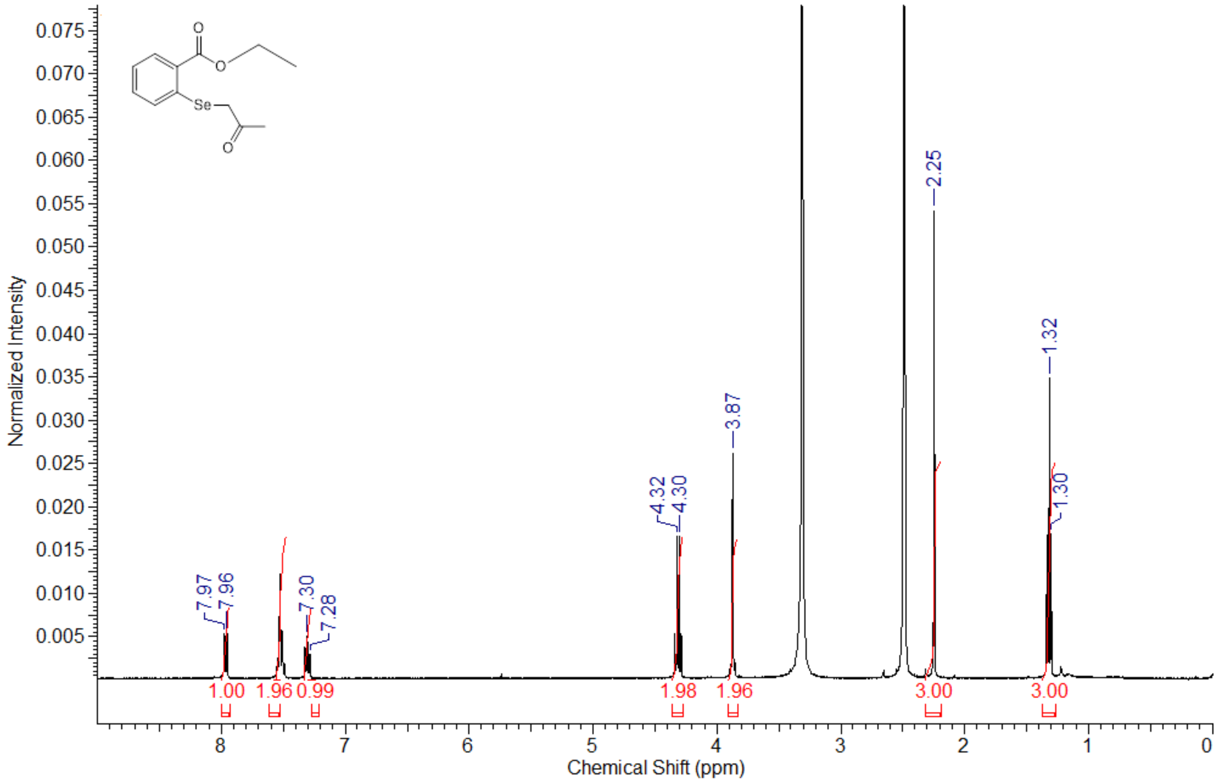

(a)

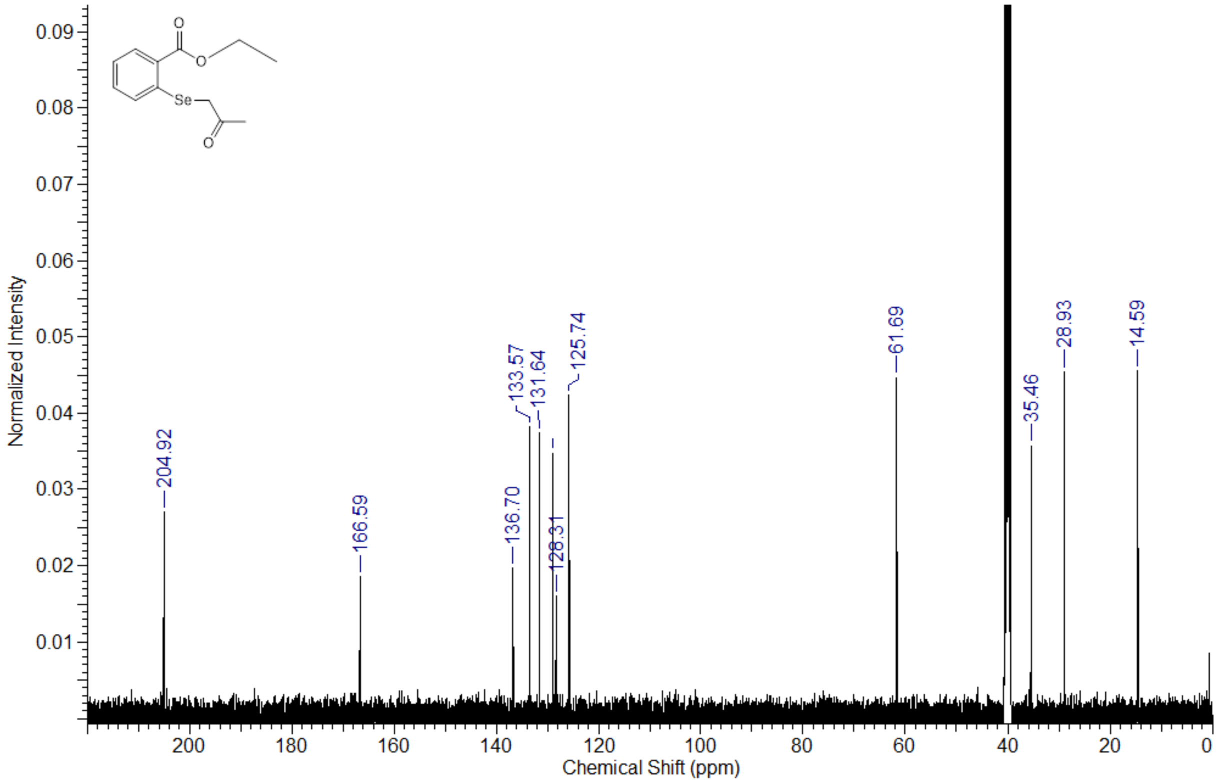

(b)

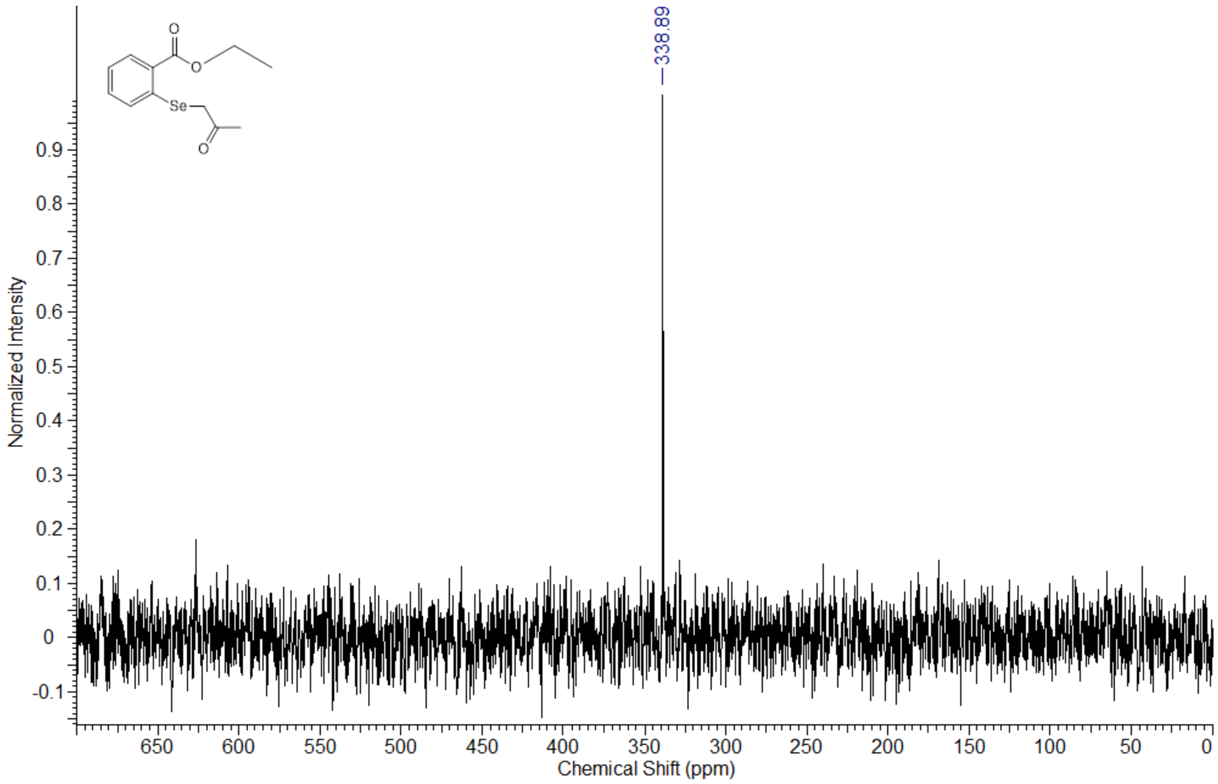

(c)

Figure S2. (**a**) 1H NMR, (**b**) 13C NMR, and (**c**) 77Se NMR spectra of *O*-(ethyl)-2-((2-oxopropyl)selanyl)benzoate **13**

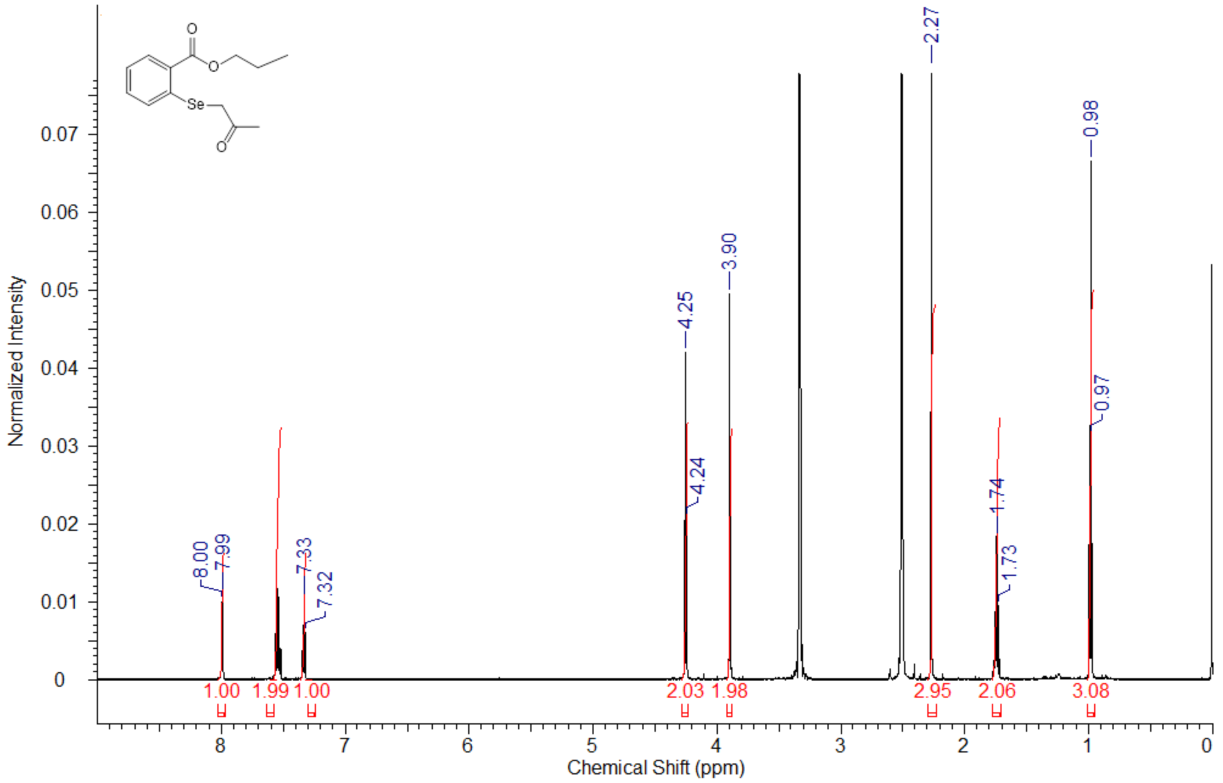

O
O
Se
O
Normalized Intensity
Chemical Shift (ppm)
0.07
0.06
0.05
0.04
0.03
0.02
0.01
0
8.00
7.99
7.33
7.32
4.25
4.24
3.90
2.27
1.74
1.73
0.98
0.97
1.00
1.99
1.00
2.03
1.98
2.95
2.06
3.08
8
7
6
5
4
3
2
1
0

(a)

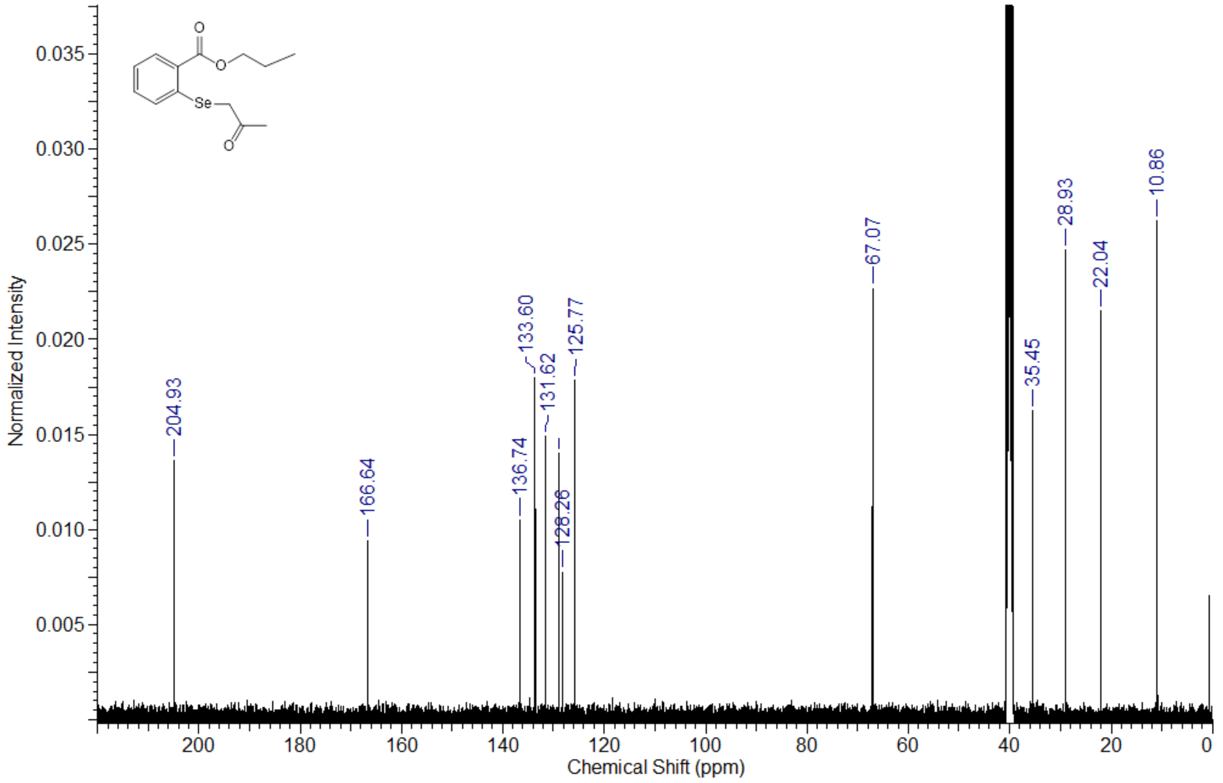

O
O
Se
O
Normalized Intensity
Chemical Shift (ppm)
0.035
0.030
0.025
0.020
0.015
0.010
0.005
204.93
166.64
136.74
133.60
131.62
128.26
125.77
67.07
35.45
28.93
22.04
10.86
200
180
160
140
120
100
80
60
40
20
0

(b)

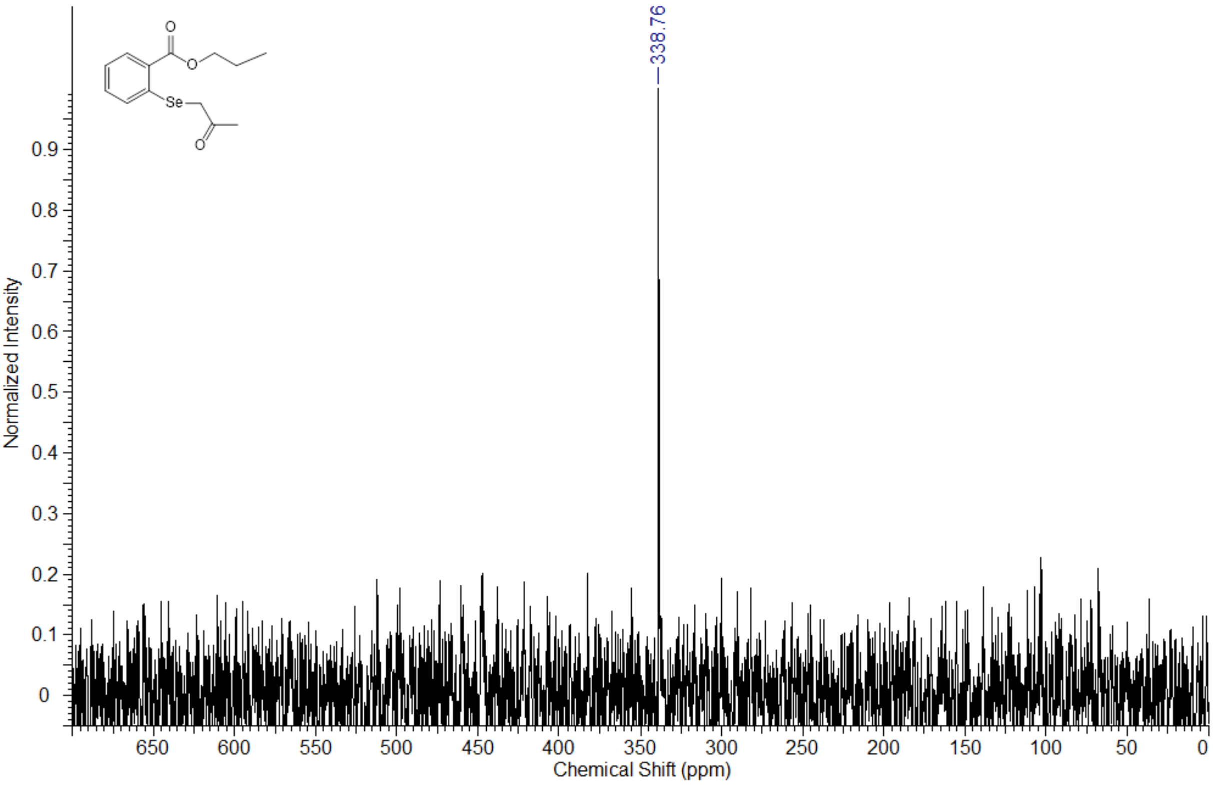

(c)

Figure S3. (**a**) 1H NMR, (**b**) 13C NMR, and (**c**) 77Se NMR spectra of *O*-(propyl)-2-((2-oxopropyl)selanyl)benzoate **14**

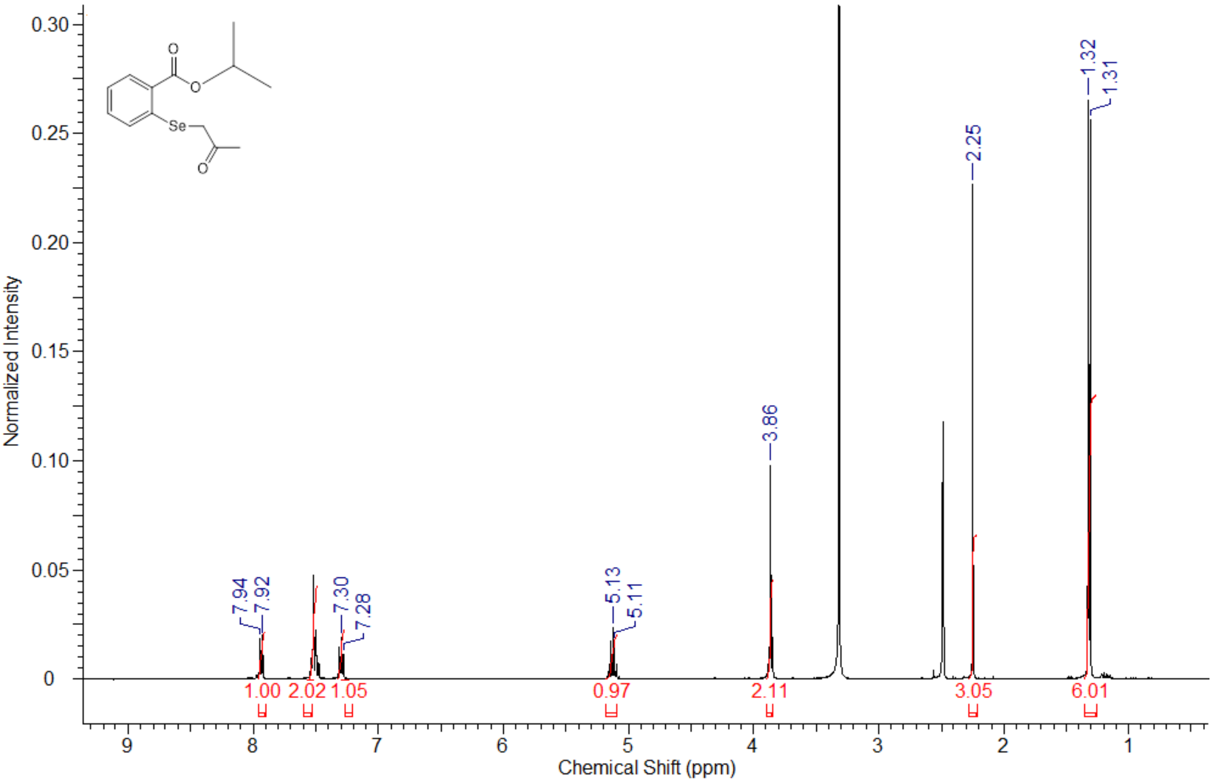

(a)

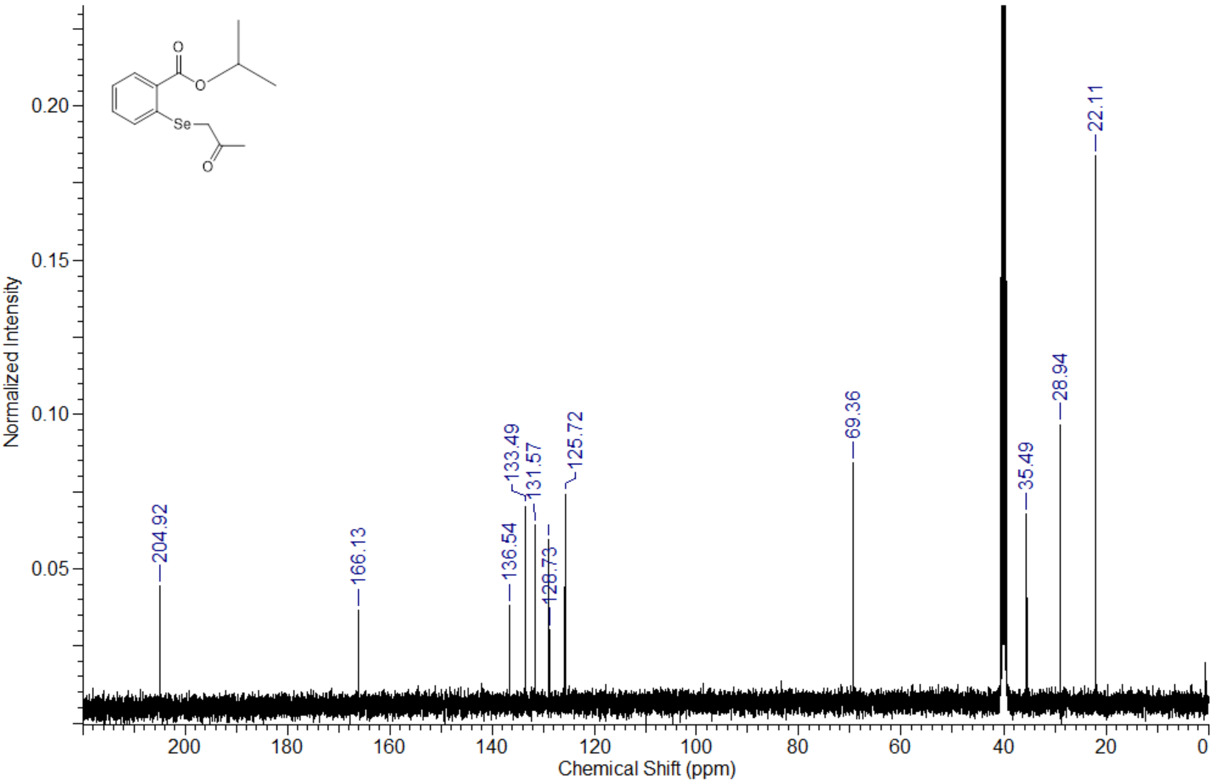

(b)

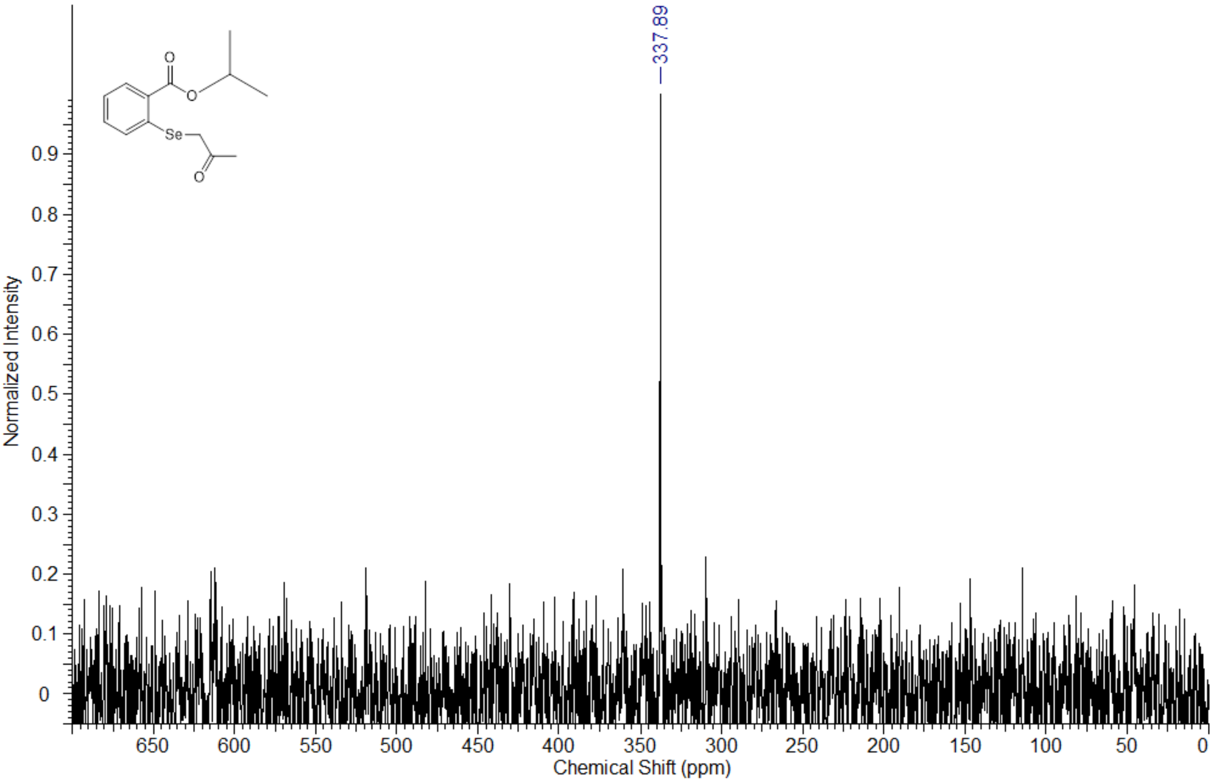

(c)

Figure S4. (**a**) 1H NMR, (**b**) 13C NMR, and (**c**) 77Se NMR spectra of *O*-(2-propyl)-2-((2-oxopropyl)selanyl)benzoate **15**

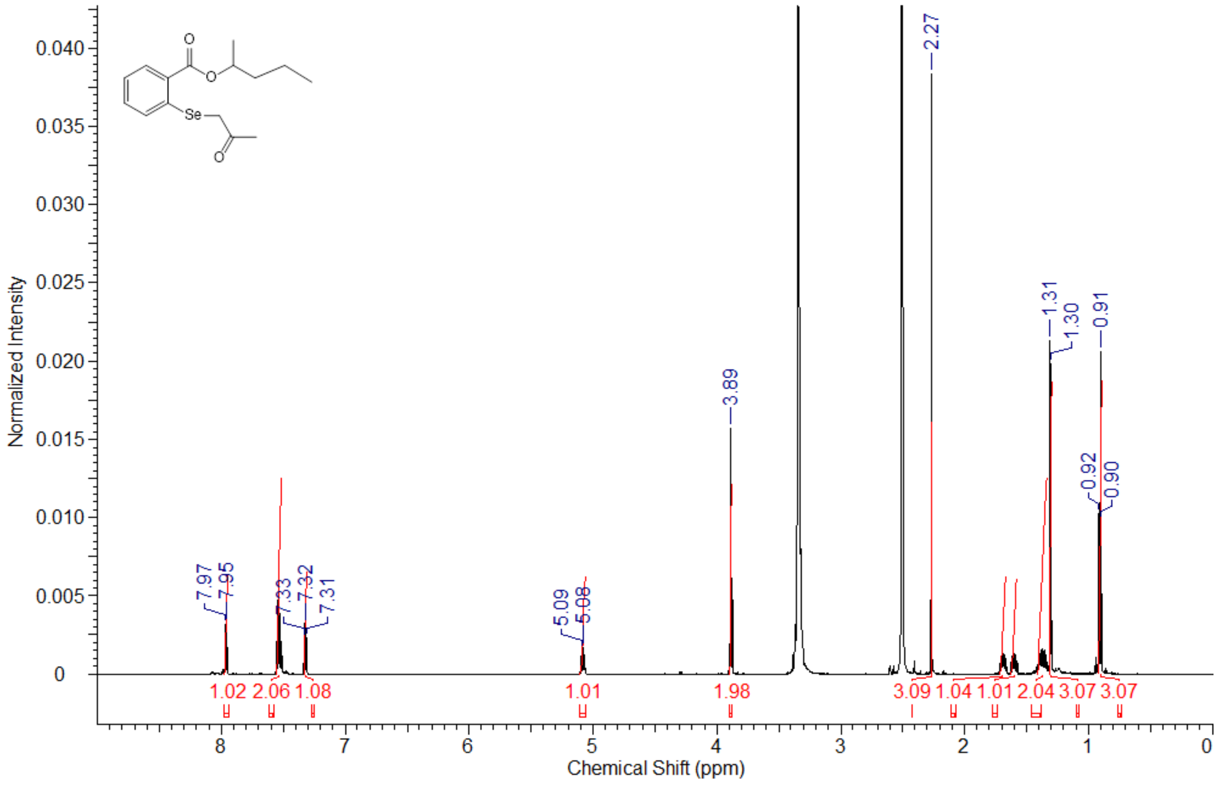
Se
Normalized Intensity
Chemical Shift (ppm)
7.97
7.95
7.33
7.32
7.31
5.09
5.08
3.89
2.27
1.31
1.30
0.92
0.91
0.90
1.02
2.06
1.08
1.01
1.98
3.09
1.04
1.01
2.04
3.07
3.07

(a)

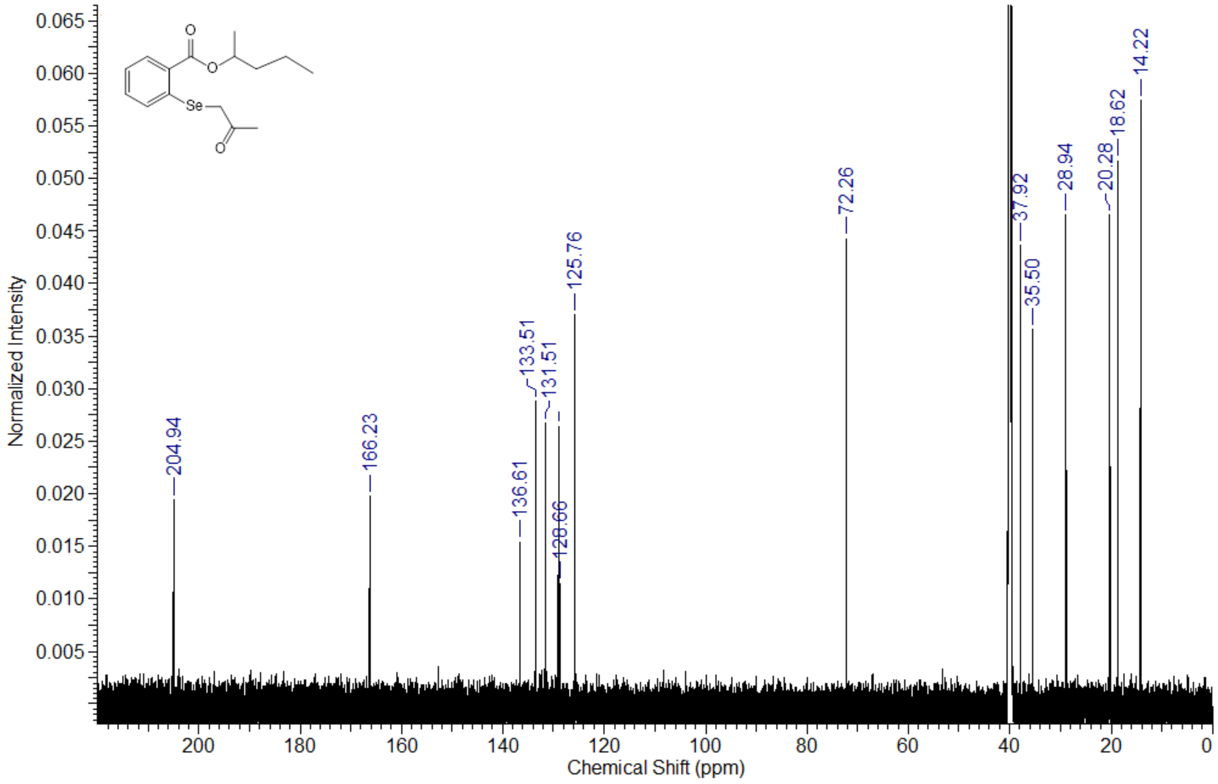
Se
Normalized Intensity
Chemical Shift (ppm)
204.94
166.23
136.61
133.51
131.51
128.66
125.76
72.26
37.92
35.50
28.94
20.28
18.62
14.22

(b)

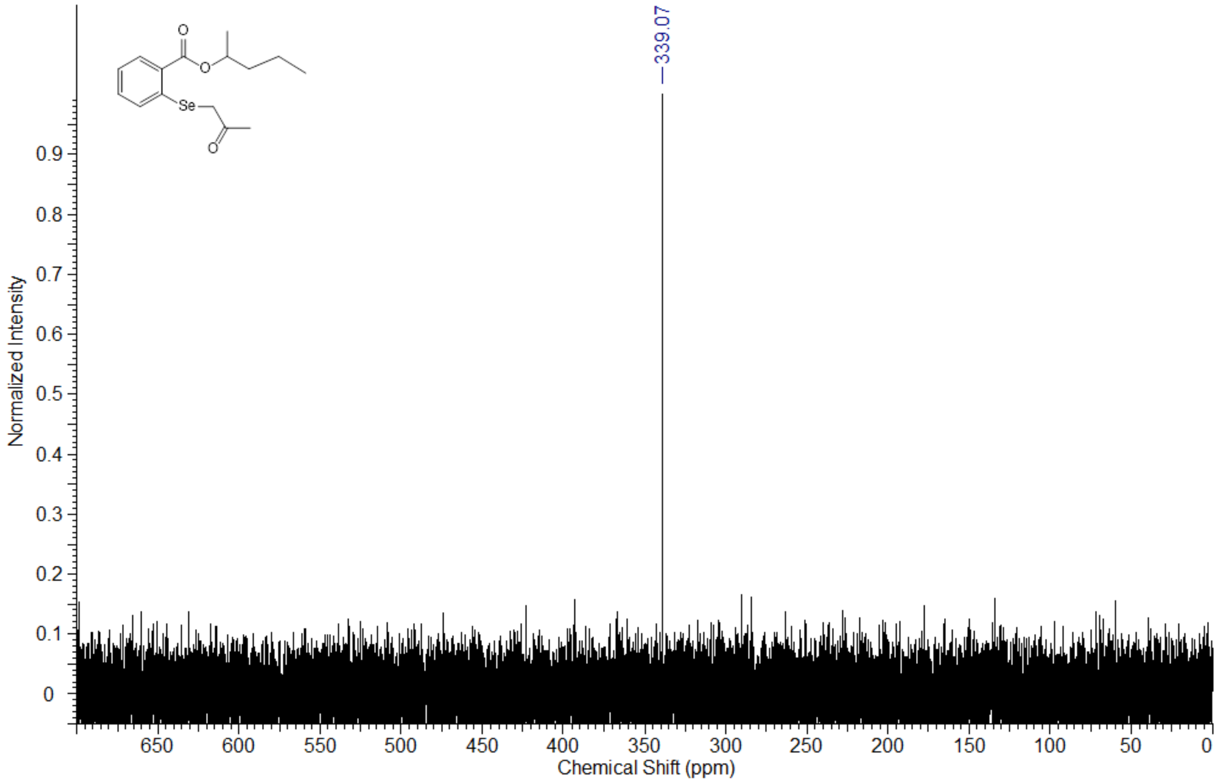

(c)

Figure S5. (**a**) 1H NMR, (**b**) 13C NMR, and (**c**) 77Se NMR spectra of *O*-(2-pentyl)-2-((2-oxopropyl)selanyl)benzoate **16**

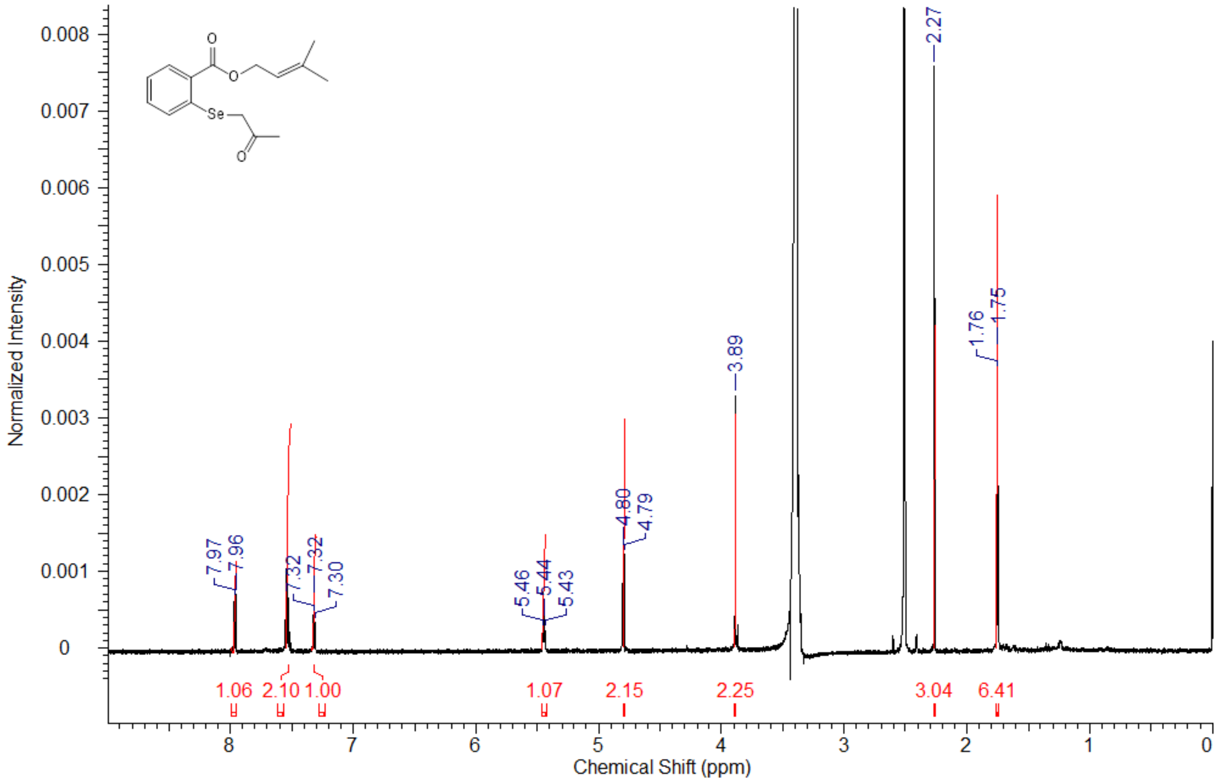

(a)

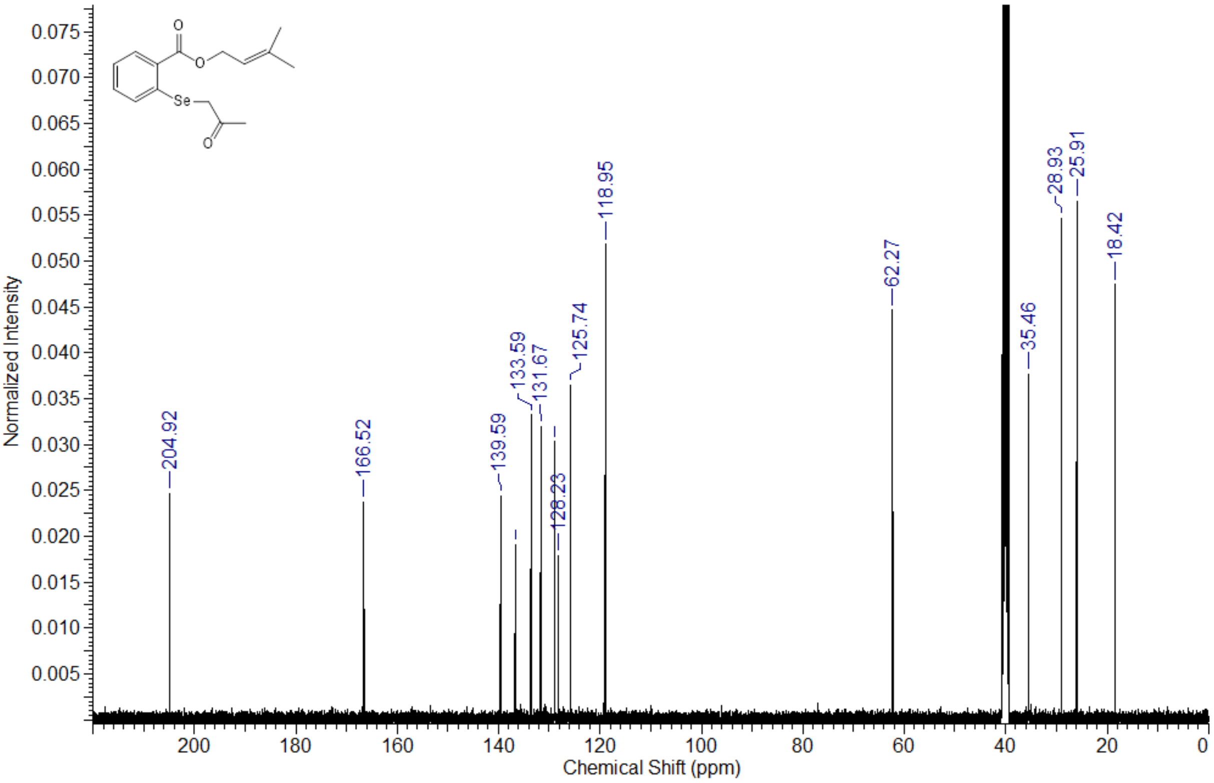

(b)

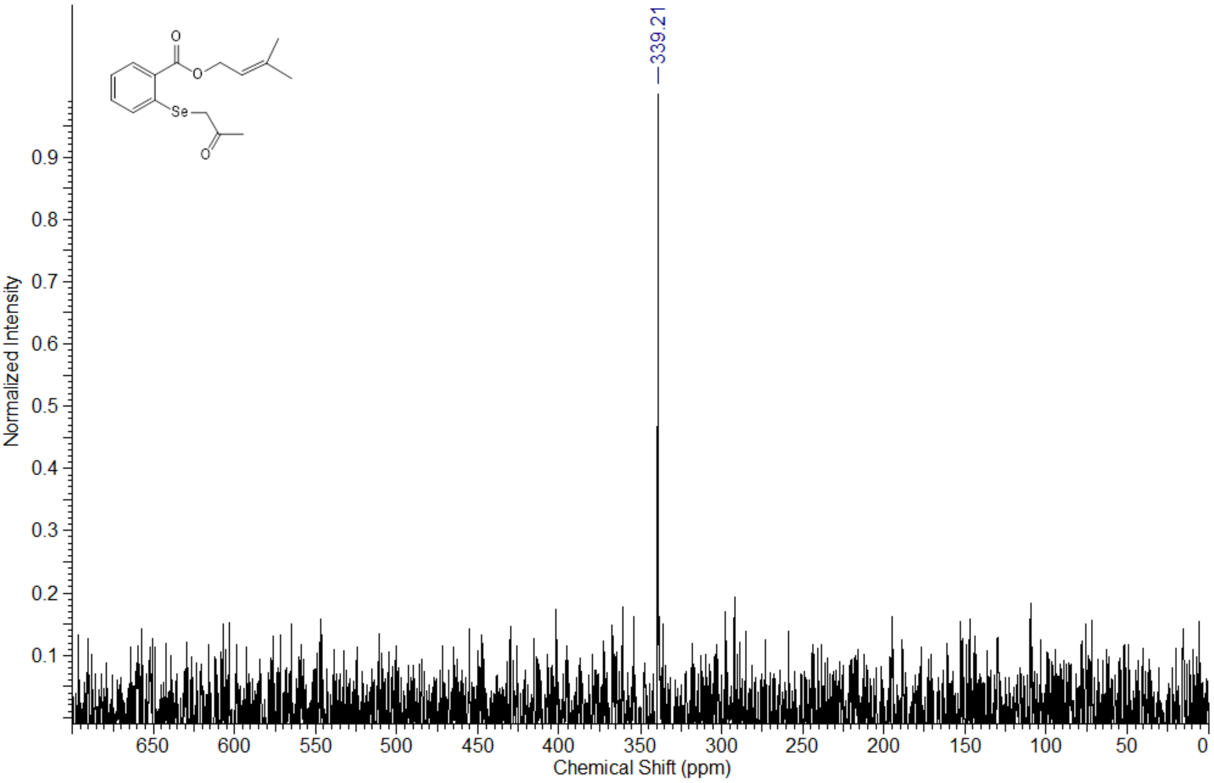

(C)

Figure S6. (**a**) 1H NMR, (**b**) 13C NMR, and (**c**) 77Se NMR spectra of *O*-(3-methylbut-2-en-1-yl)-2-((2-oxopropyl)selanyl)benzoate **17**

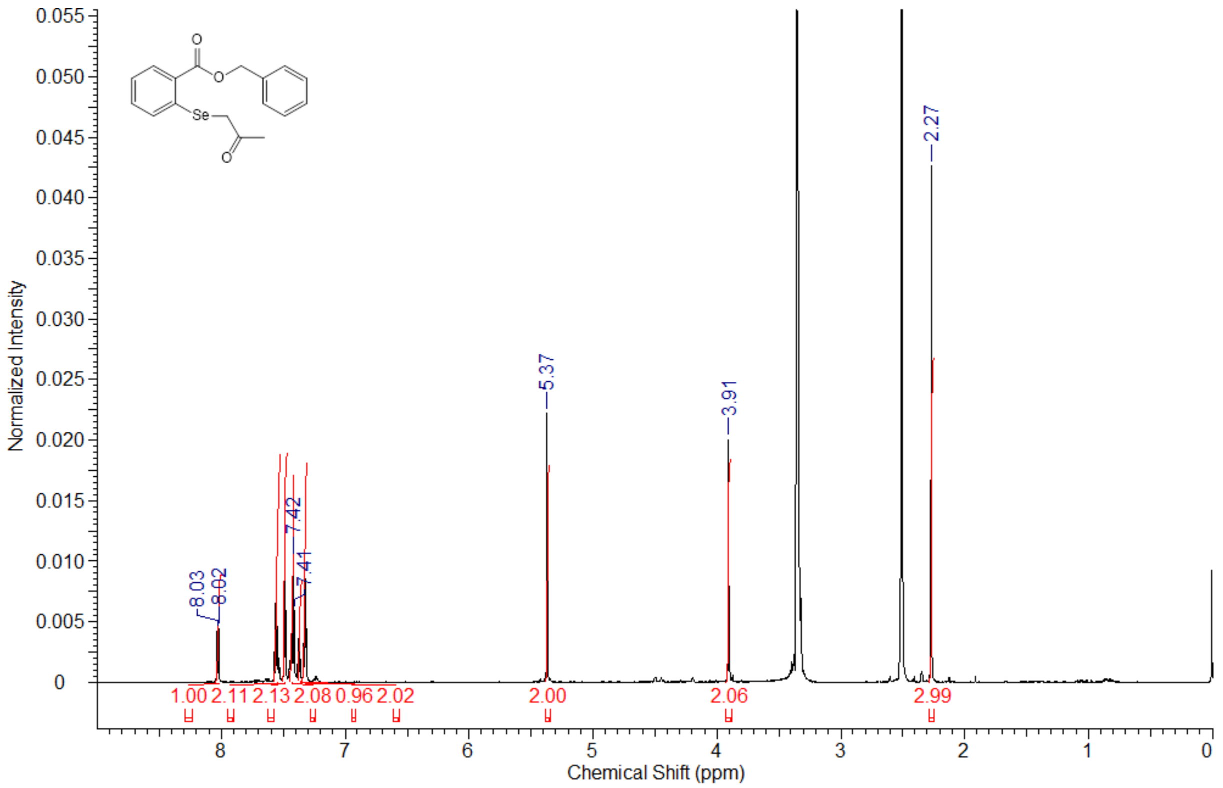
Normalized Intensity
Chemical Shift (ppm)
Se
O
8.03
8.02
7.42
7.41
5.37
3.91
2.27
1.00 2.11 2.13 2.08 0.96 2.02
2.00
2.06
2.99

(a)

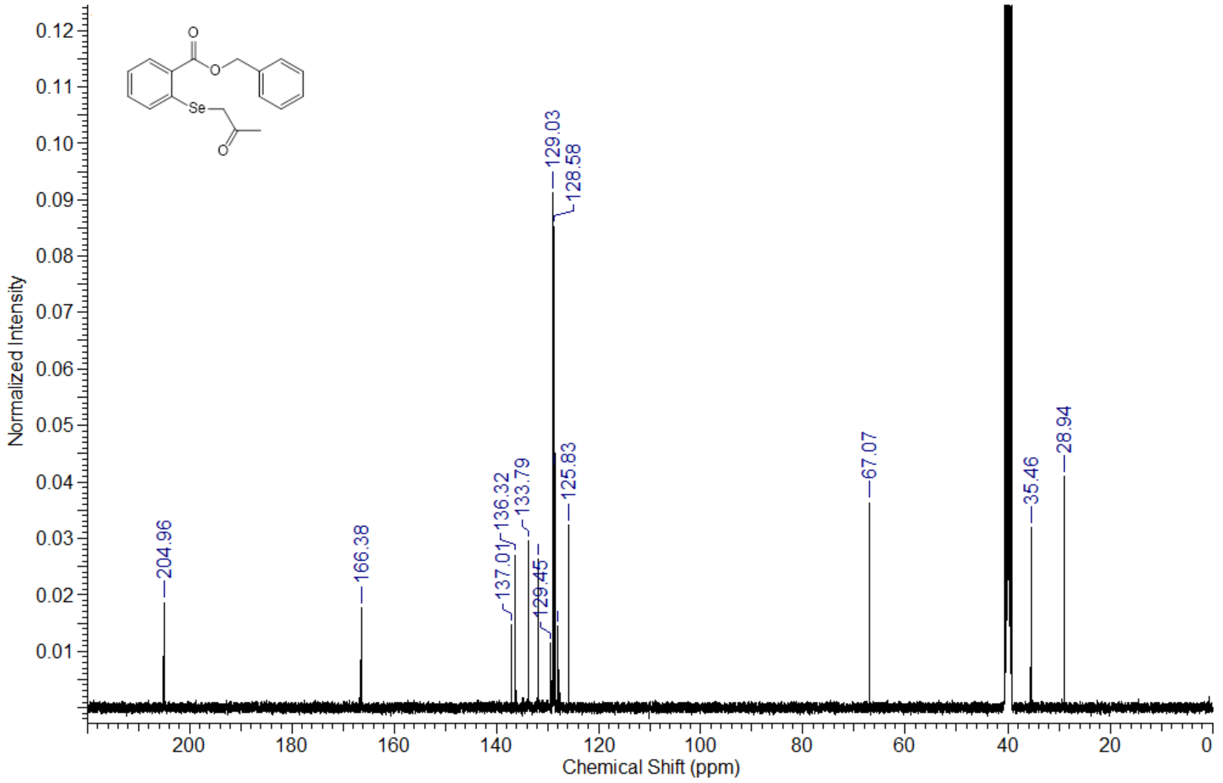
Normalized Intensity
Chemical Shift (ppm)
Se
O
204.96
166.38
137.01
136.32
133.79
129.45
129.03
128.58
125.83
67.07
35.46
28.94

(b)

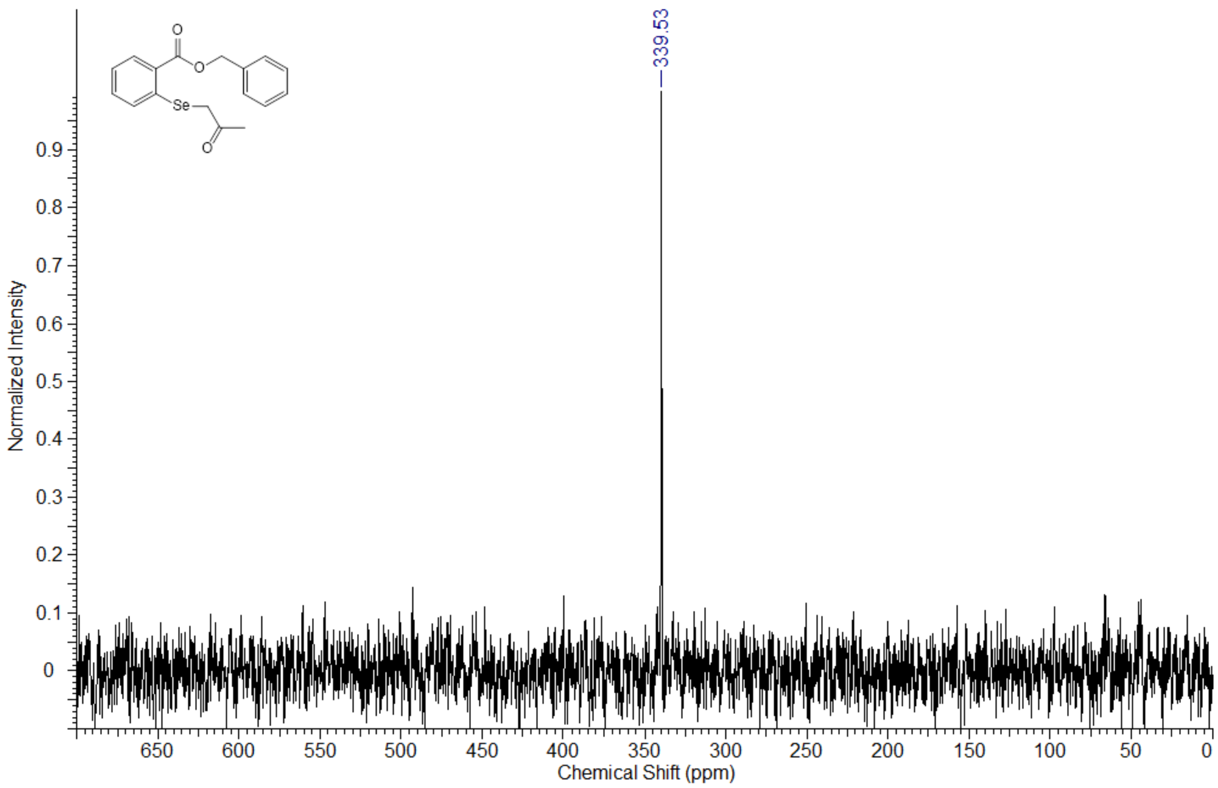

(c)

Figure S7. (**a**) 1H NMR, (**b**) 13C NMR, and (**c**) 77Se NMR spectra of *O*-(benzyl)-2-((2-oxopropyl)selanyl)benzoate **18**

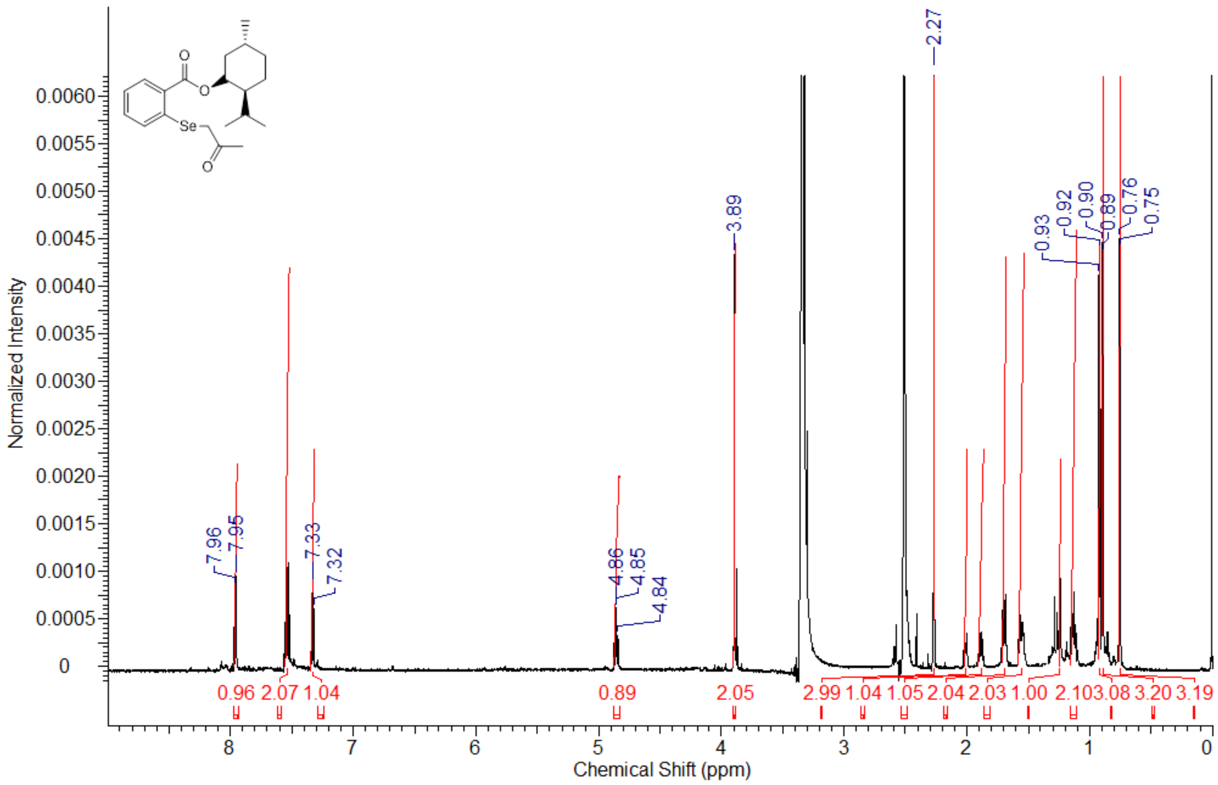

(a)

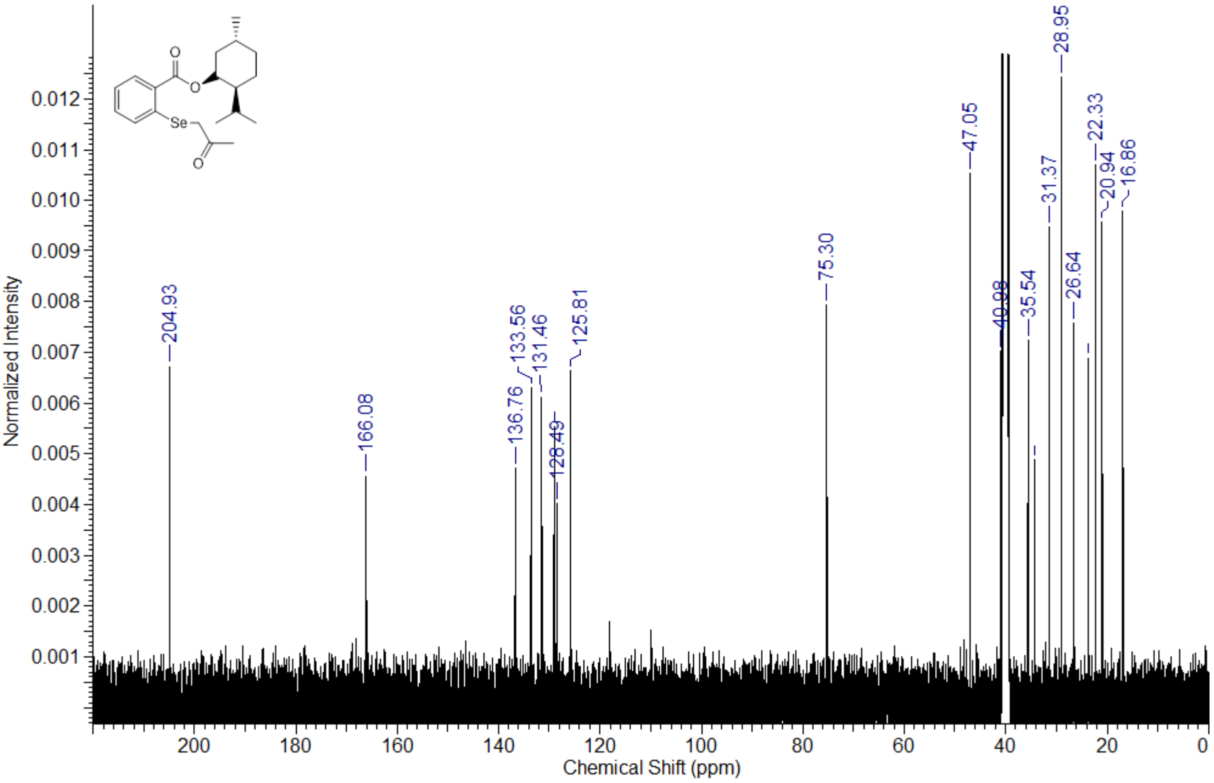

(b)

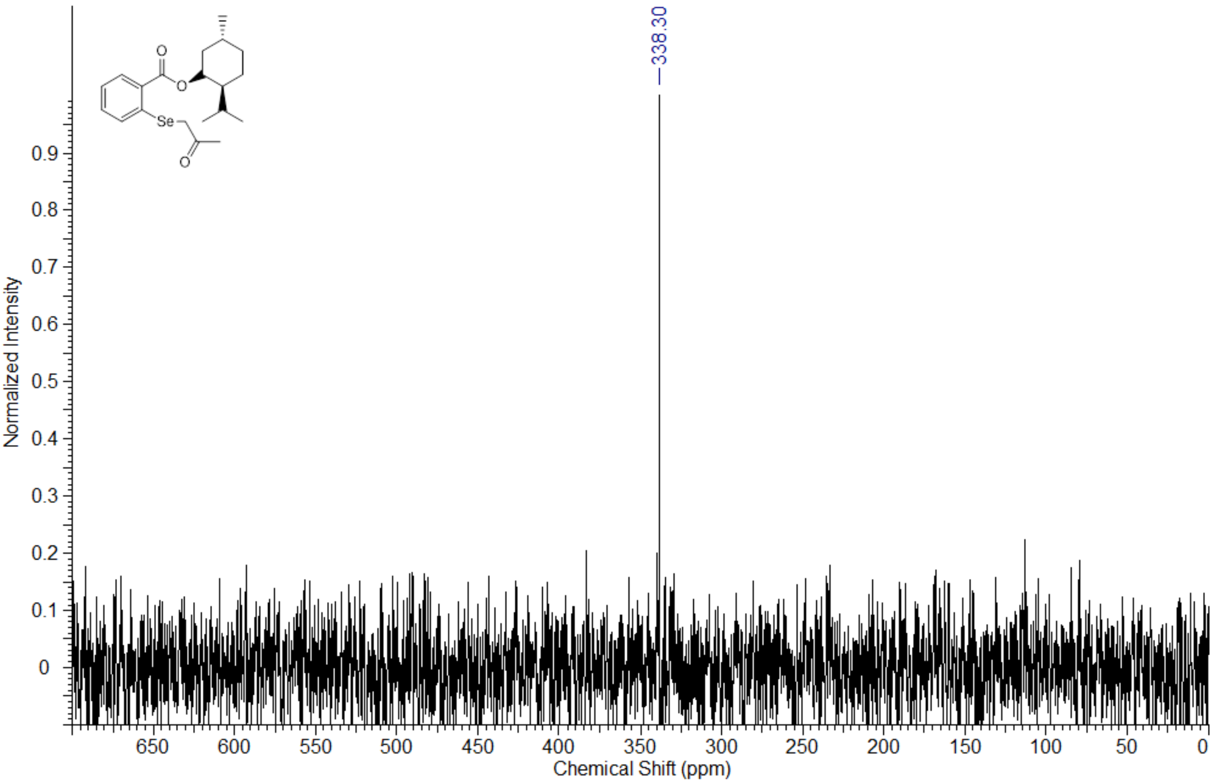

(c)

Figure S8. (**a**) 1H NMR, (**b**) 13C NMR, and (**c**) 77Se NMR spectra of *O*-((1*R*,2*S*,5*R*)-(-)-2-isopropyl-5-methylcyclohexyl)-2-((2-oxopropyl)selanyl)benzoate **19**

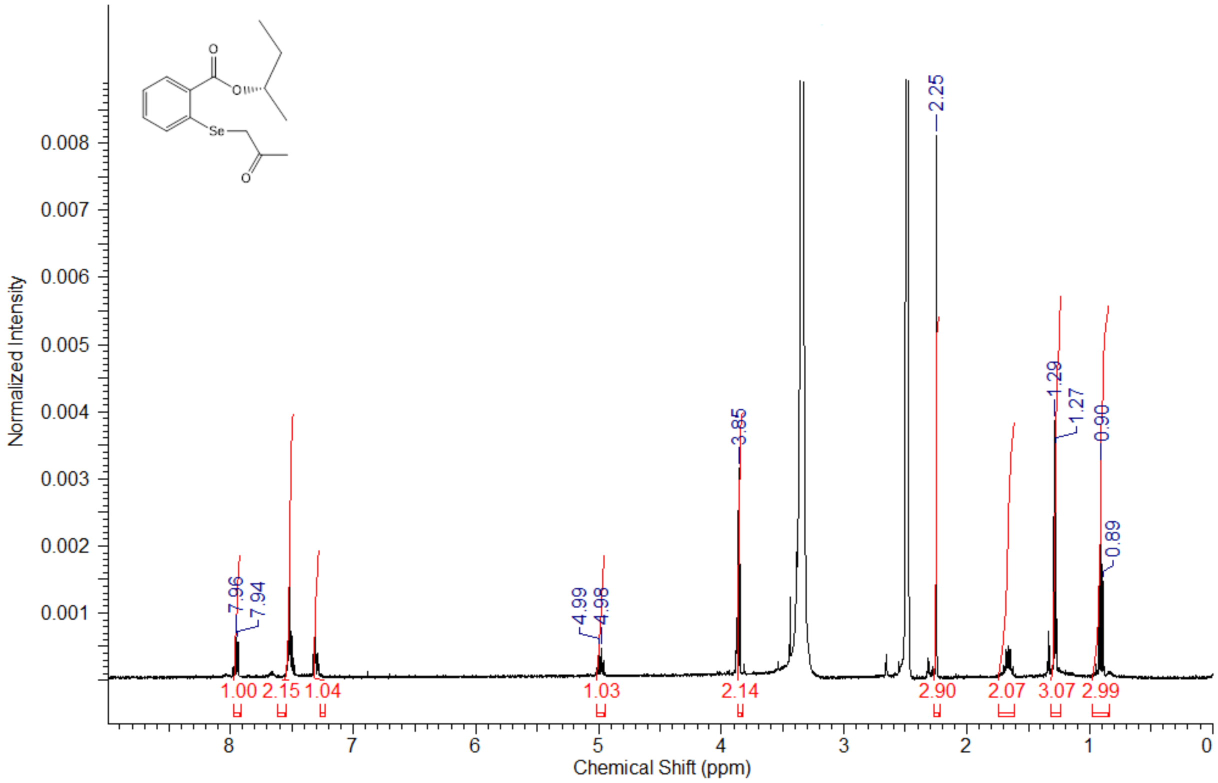

0.008
0.007
0.006
0.005
0.004
0.003
0.002
0.001
Normalized Intensity
7.96
7.94
4.99
4.98
3.85
2.25
1.29
1.27
0.90
0.89
1.00
2.15
1.04
1.03
2.14
2.90
2.07
3.07
2.99
8
7
6
5
4
3
2
1
0
Chemical Shift (ppm)

(a)

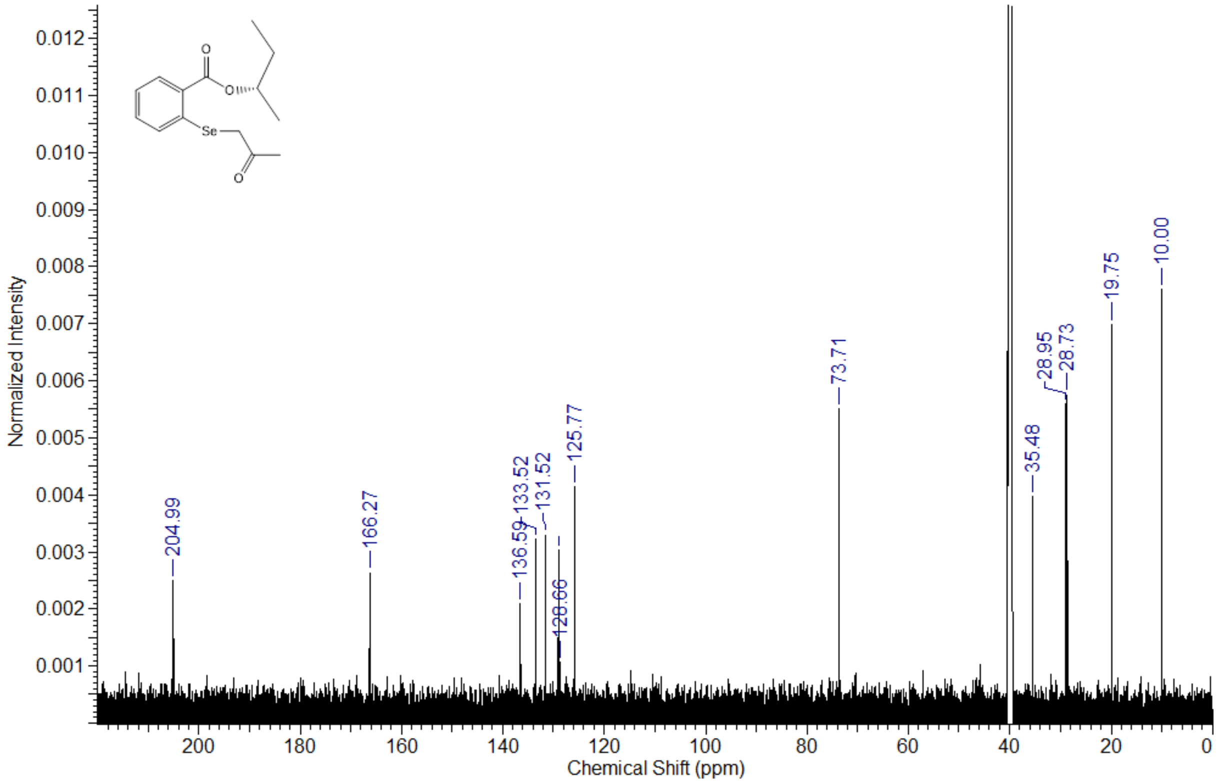

0.012
0.011
0.010
0.009
0.008
0.007
0.006
0.005
0.004
0.003
0.002
0.001
Normalized Intensity
204.99
166.27
136.59
133.52
131.52
128.66
125.77
73.71
35.48
28.95
28.73
19.75
10.00
200
180
160
140
120
100
80
60
40
20
0
Chemical Shift (ppm)

(b)

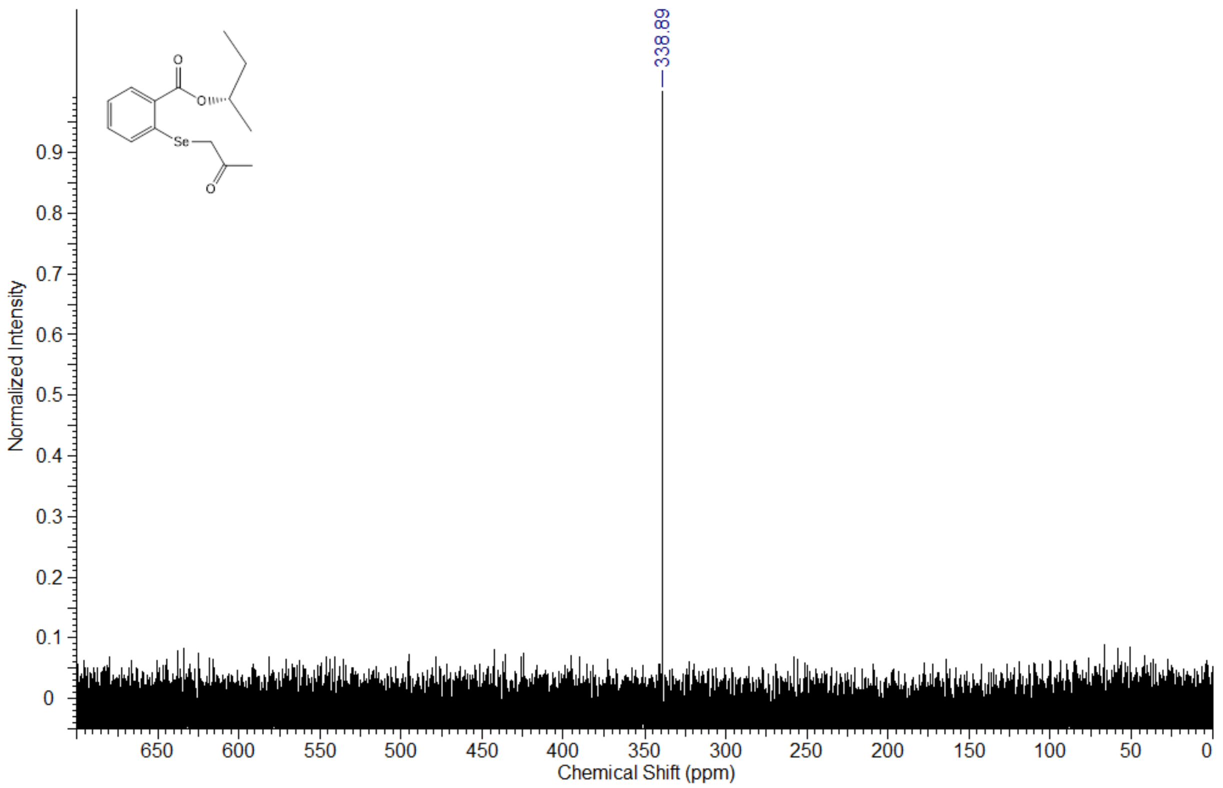

(c)

Figure S9. (**a**) 1H NMR, (**b**) 13C NMR, and (**c**) 77Se NMR spectra of *O*-((*S*)-(+)-*sec*-butyl)-2-((2-oxopropyl)selanyl)benzoate **20**

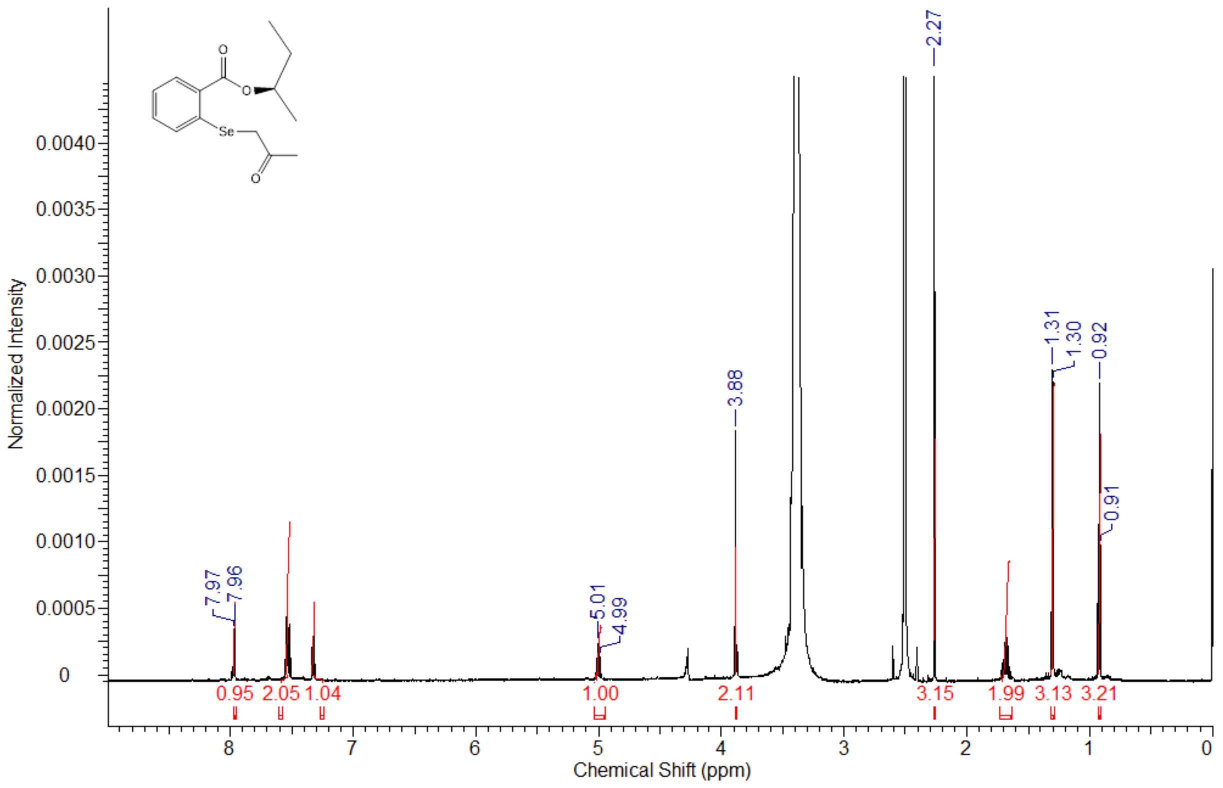

(a)

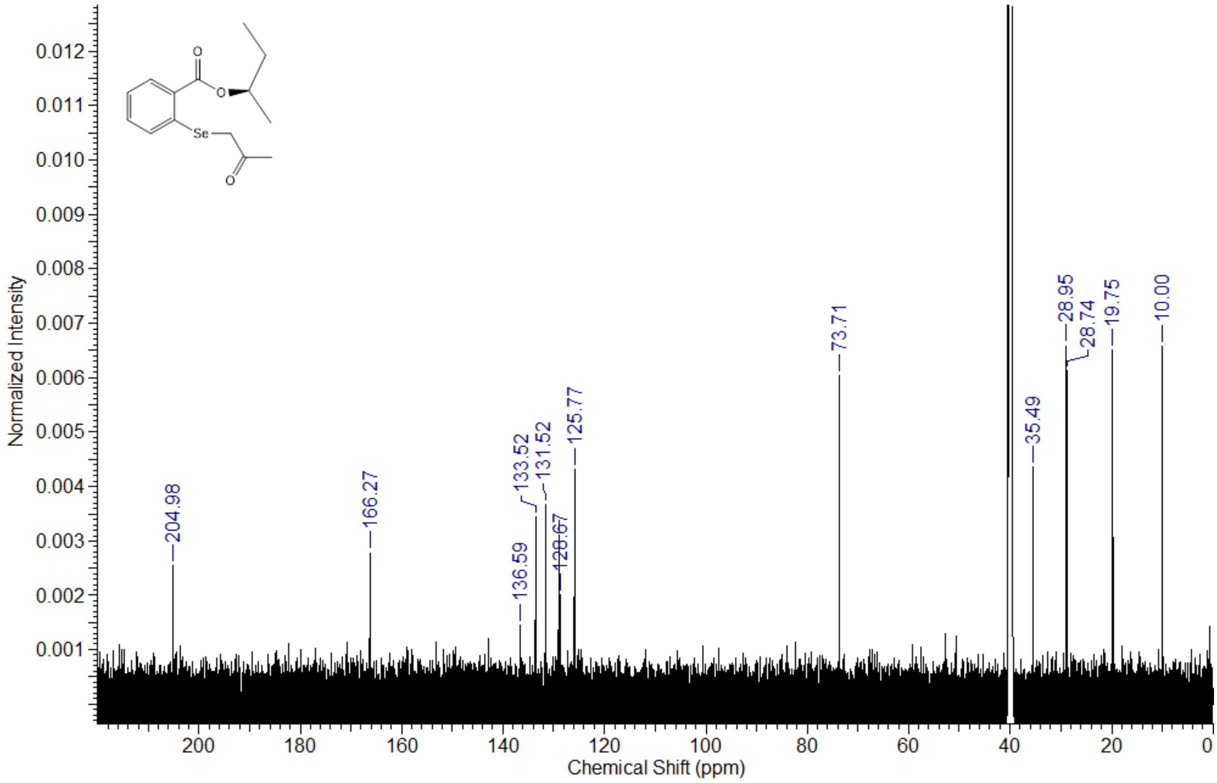

(b)

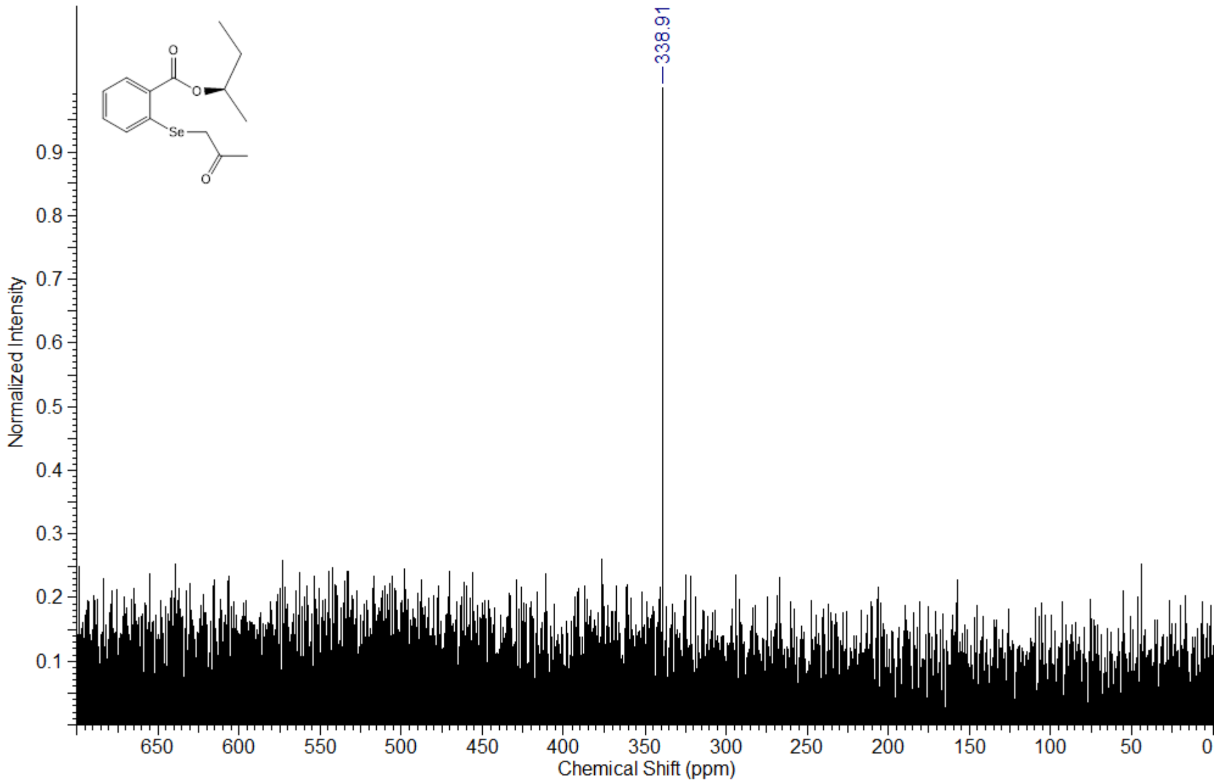

(c)

Figure 10. (**a**) 1H NMR, (**b**) 13C NMR, and (**c**) 77Se NMR spectra of *O*-((*R*)-(-)-*sec*-butyl)-2-((2-oxopropyl)selanyl)benzoate **21**

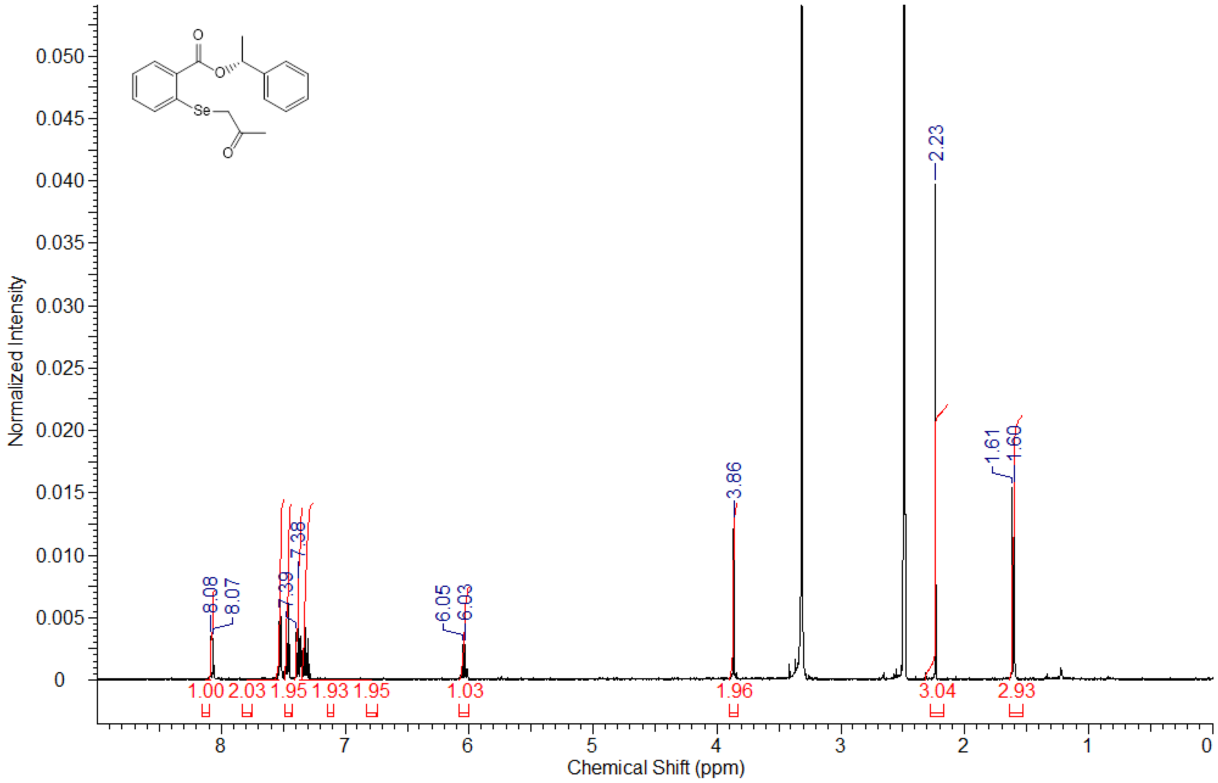
Normalized Intensity
Chemical Shift (ppm)
O
O
Se
O
8.08
8.07
7.39
7.38
6.05
6.03
3.86
2.23
1.61
1.60
1.00
2.03
1.95
1.93
1.95
1.03
1.96
3.04
2.93

(a)

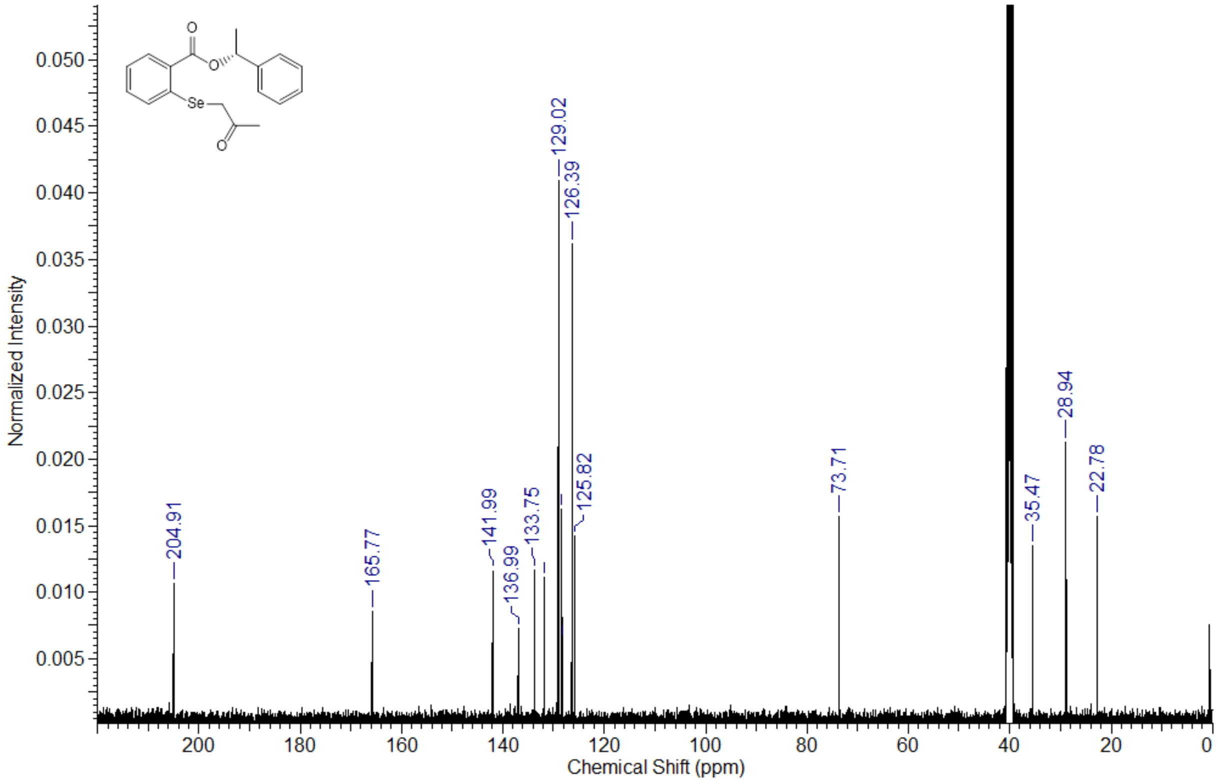
Normalized Intensity
Chemical Shift (ppm)
O
O
Se
O
204.91
165.77
141.99
136.99
133.75
129.02
126.39
125.82
73.71
35.47
28.94
22.78

(b)

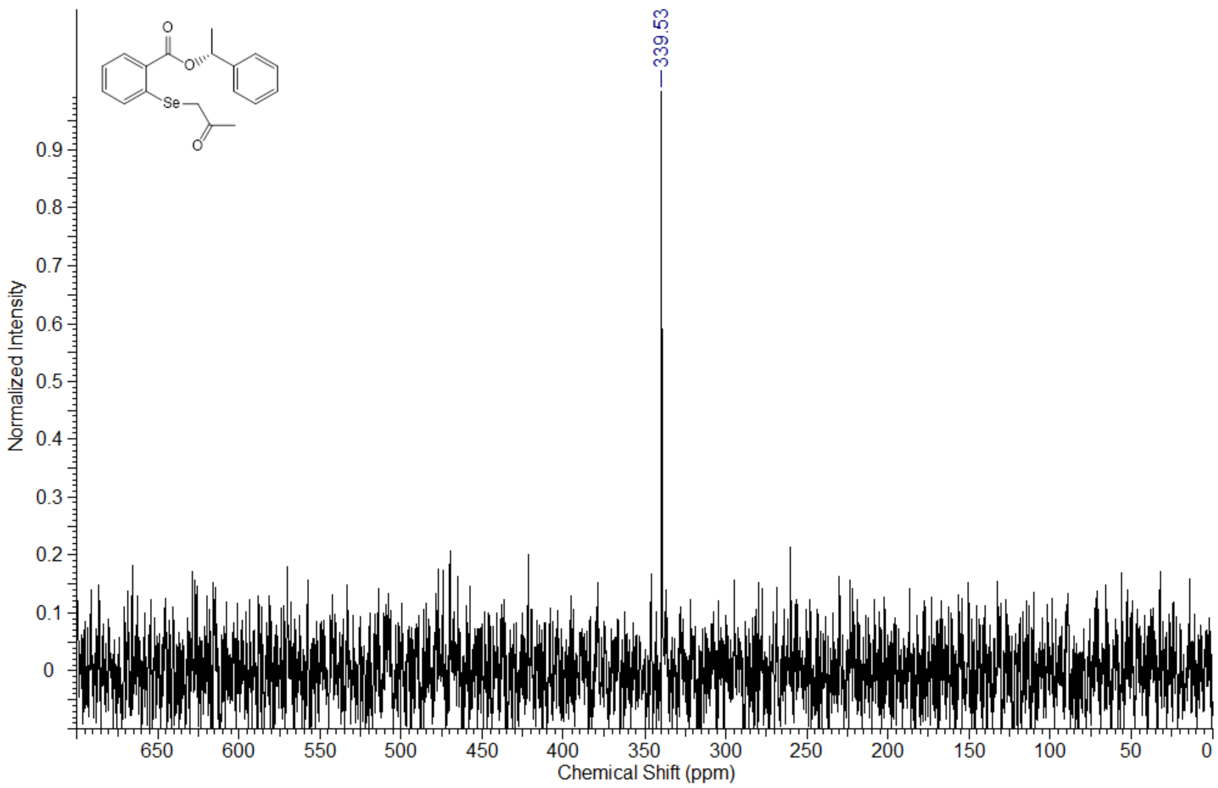

(c)

Figure S11. (**a**) 1H NMR, (**b**) 13C NMR, and (**c**) 77Se NMR spectra of *O*-((*R*)-(+)-α-methylbenzyl)-2-((2-oxopropyl)selanyl)benzoate **10**

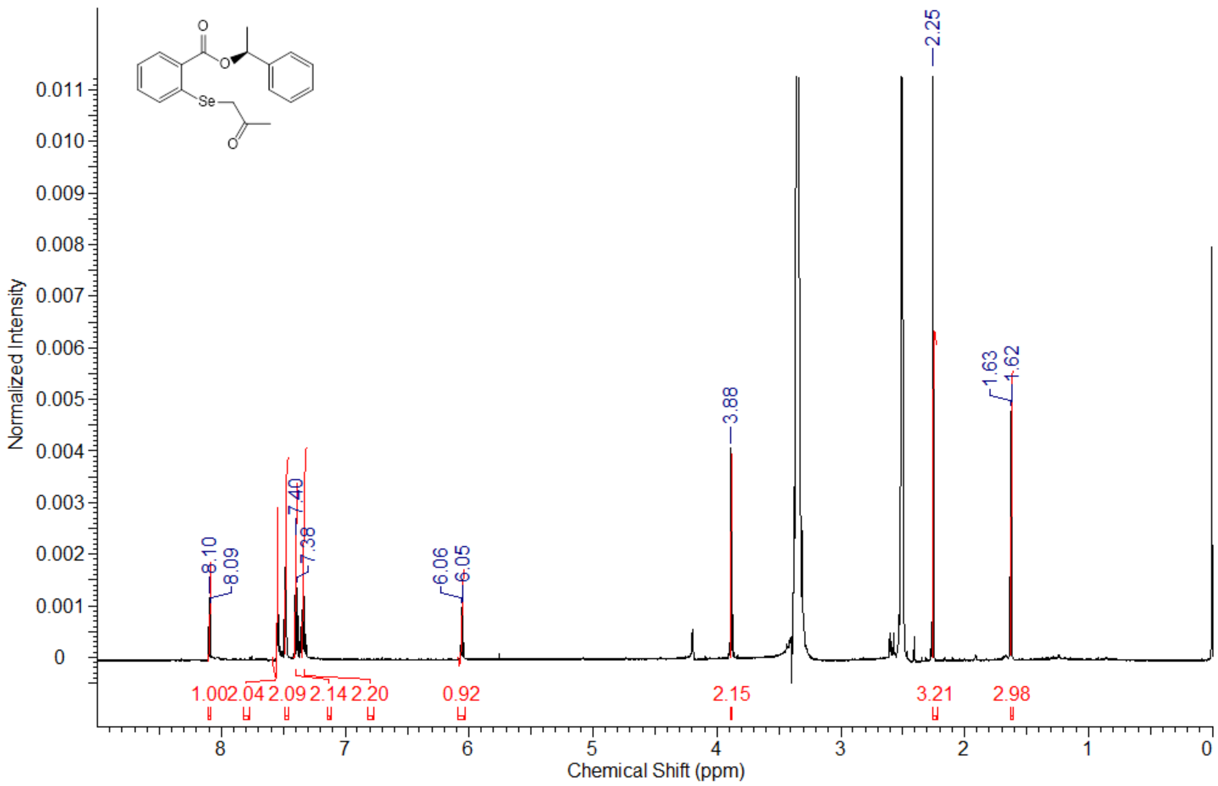

(a)

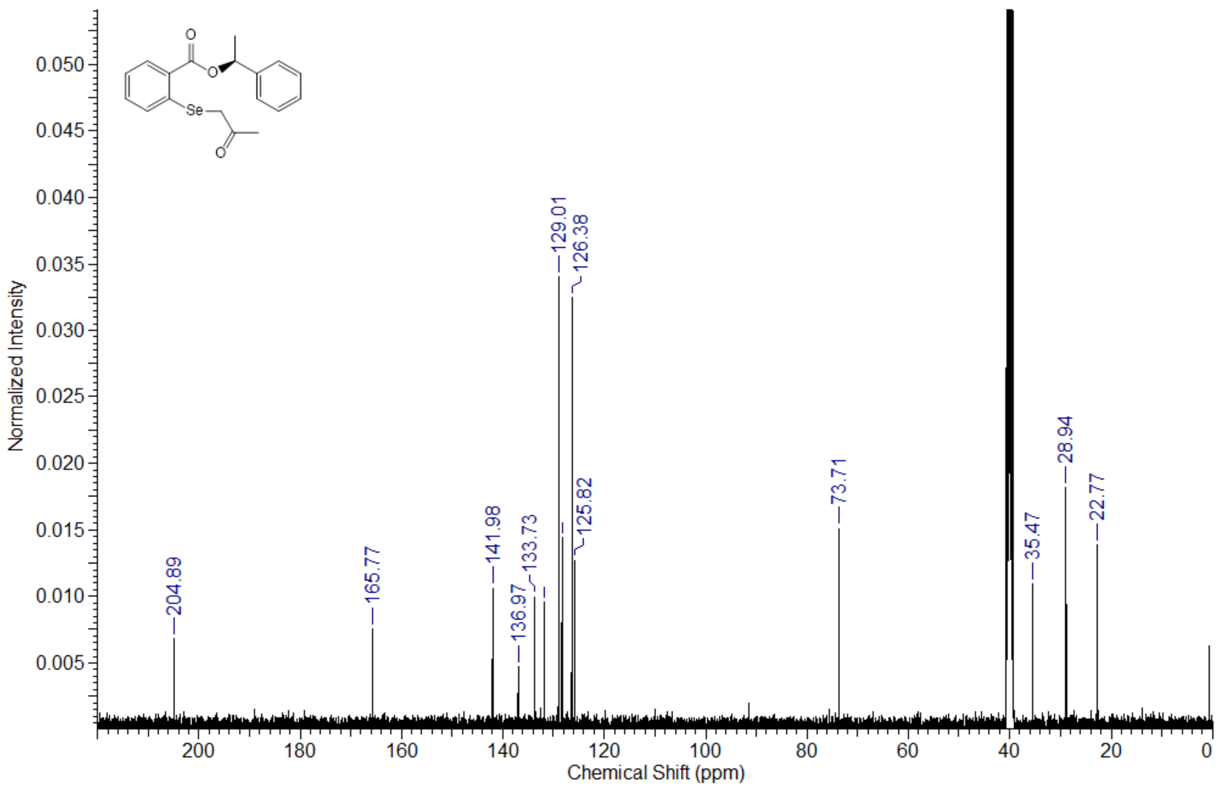

(b)

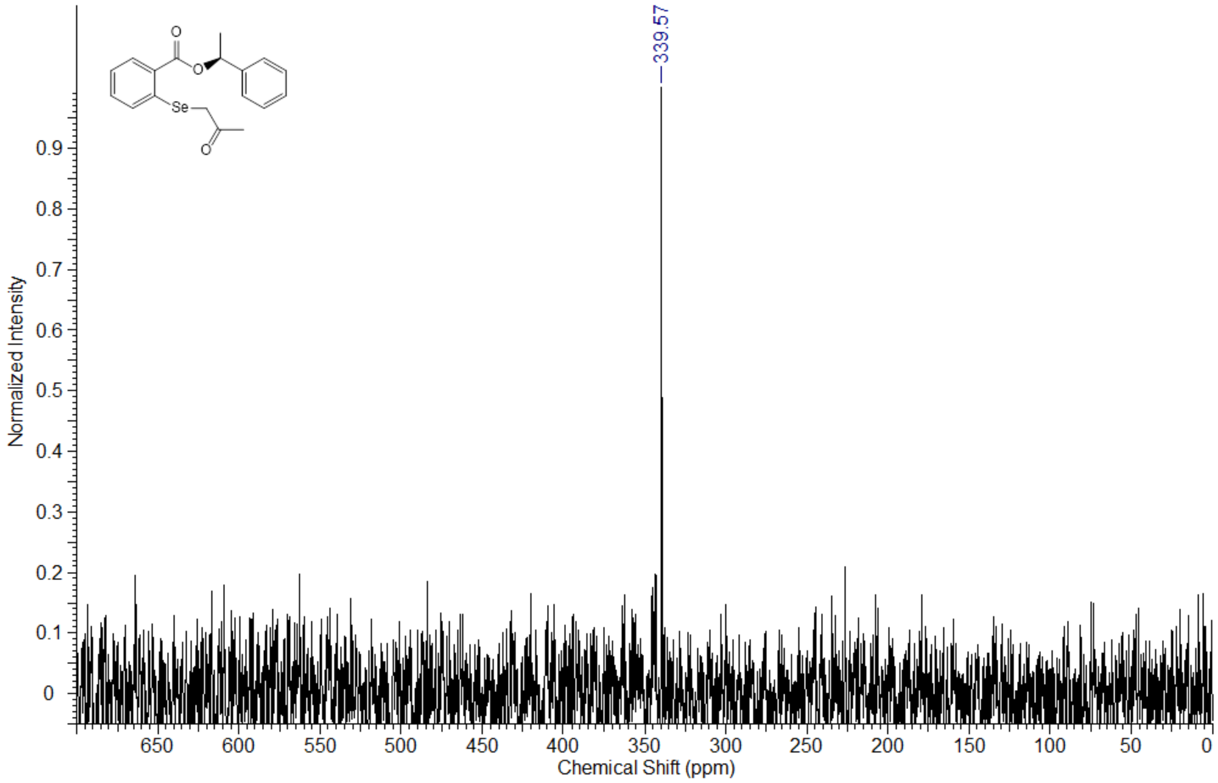

(c)

Figure S12. (**a**) 1H NMR, (**b**) 13C NMR, and (**c**) 77Se NMR spectra of *O*-((*S*)-(-)-α-methylbenzyl)-2-((2-oxopropyl)selanyl)benzoate **22**

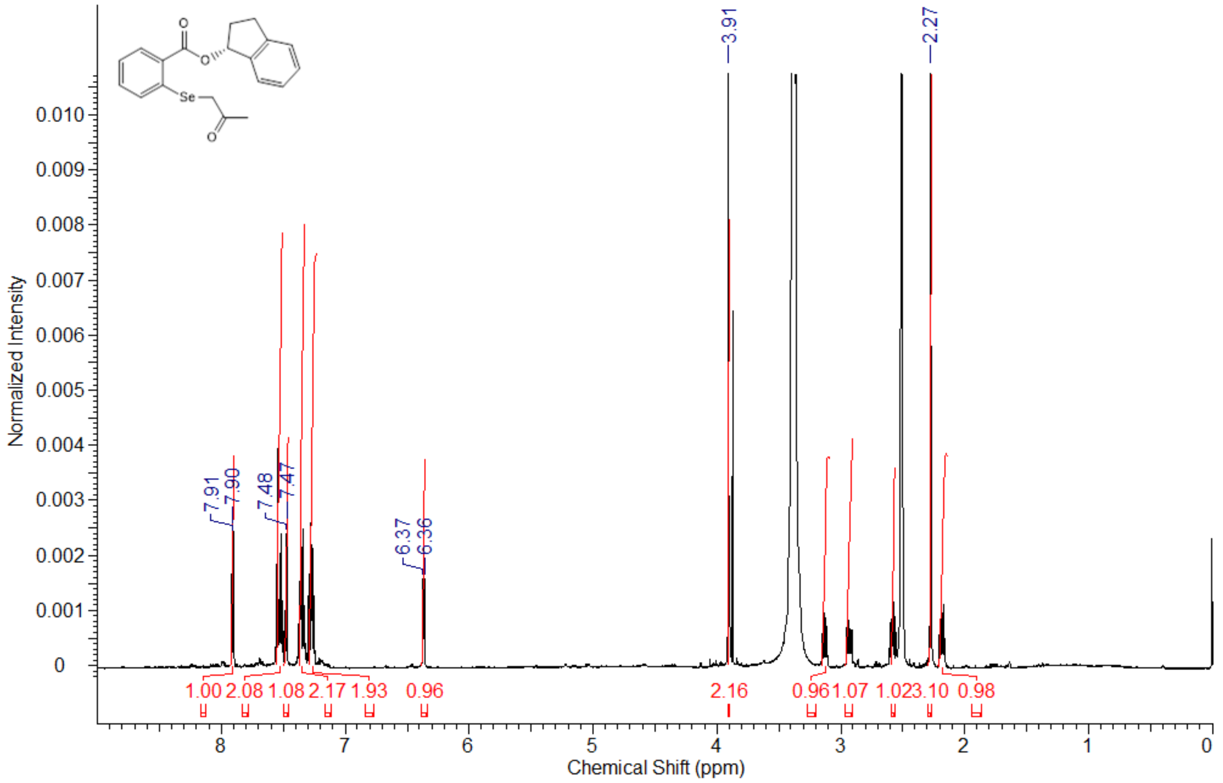

Normalized Intensity
Chemical Shift (ppm)
7.91
7.90
7.48
7.47
6.37
6.36
3.91
2.27
1.00 2.08 1.08 2.17 1.93
0.96
2.16
0.96 1.07 1.02 3.10 0.98
Se

(a)

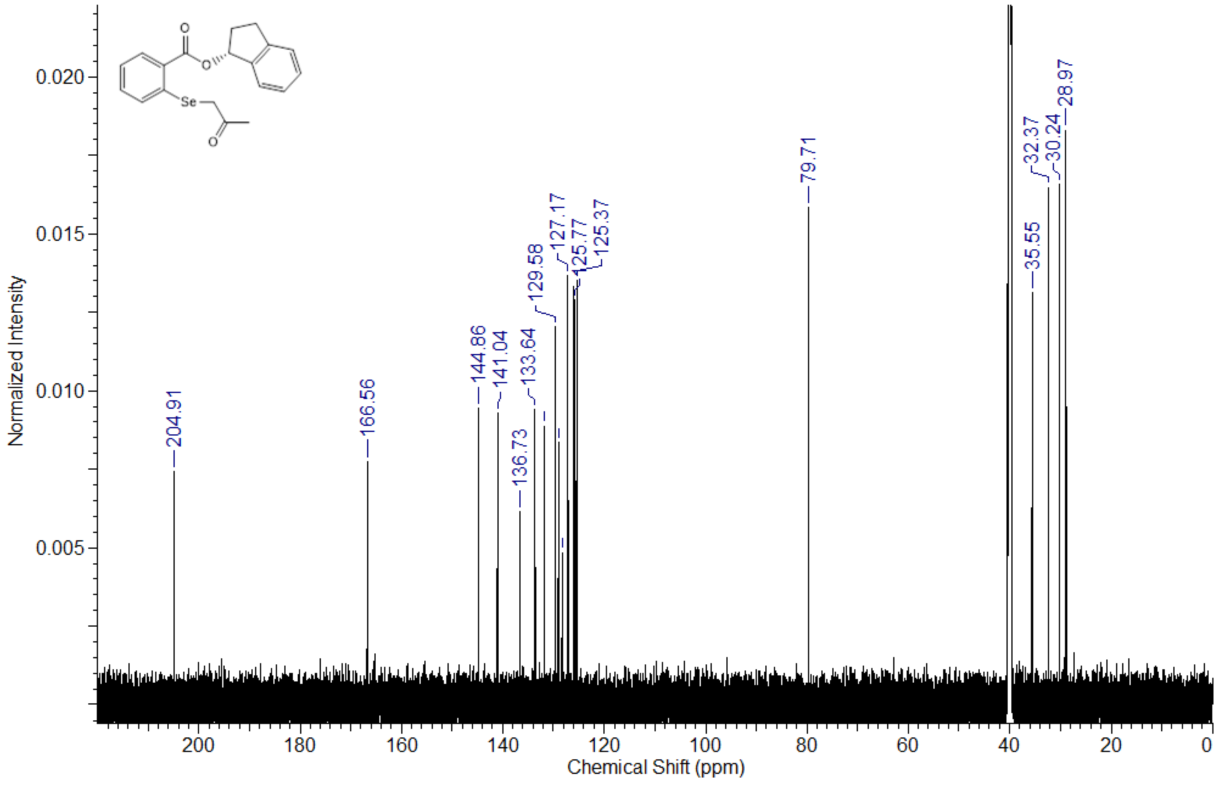

Normalized Intensity
Chemical Shift (ppm)
204.91
166.56
144.86
141.04
136.73
133.64
129.58
127.17
125.77
125.37
79.71
35.55
32.37
30.24
28.97
Se

(b)

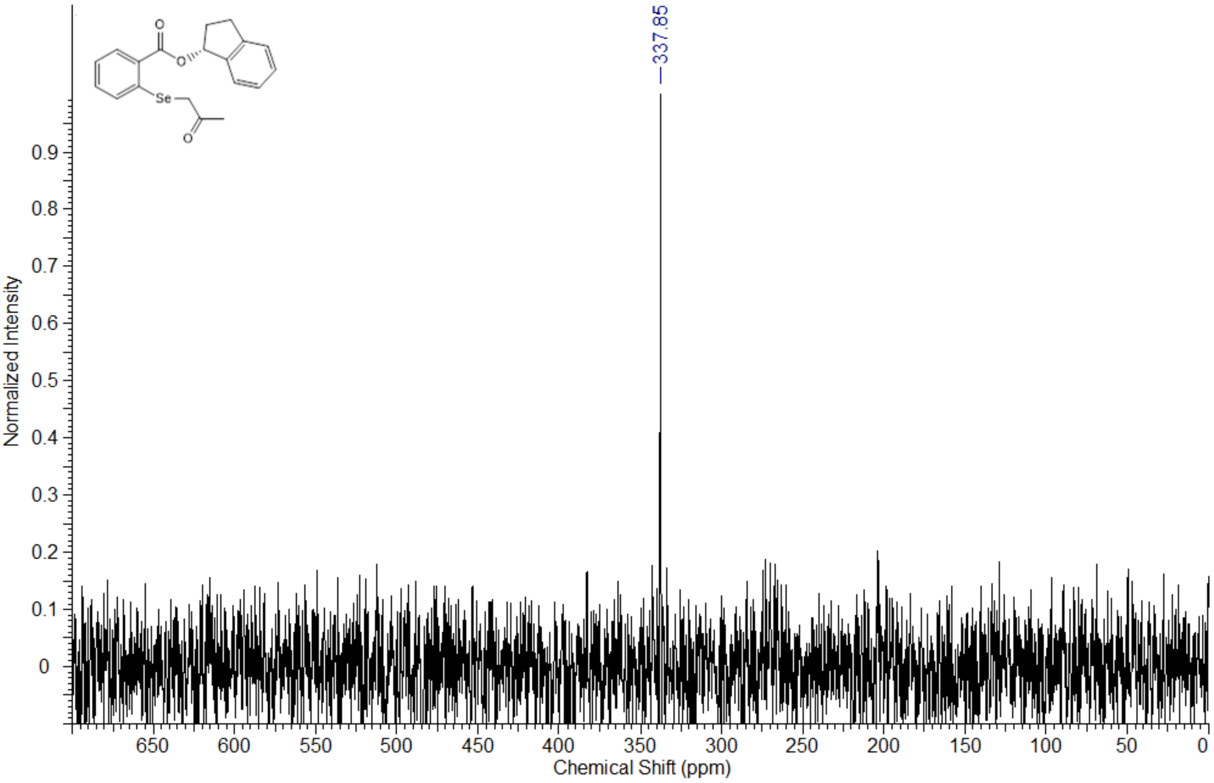

(c)

Figure S13. (**a**) 1H NMR, (**b**) 13C NMR, and (**c**) 77Se NMR spectra of *O*-((*R*)-(-)-2,3-dihydro-(1*H)*-inden-1-yl)-2-((2-oxopropyl)selanyl)benzoate **23**

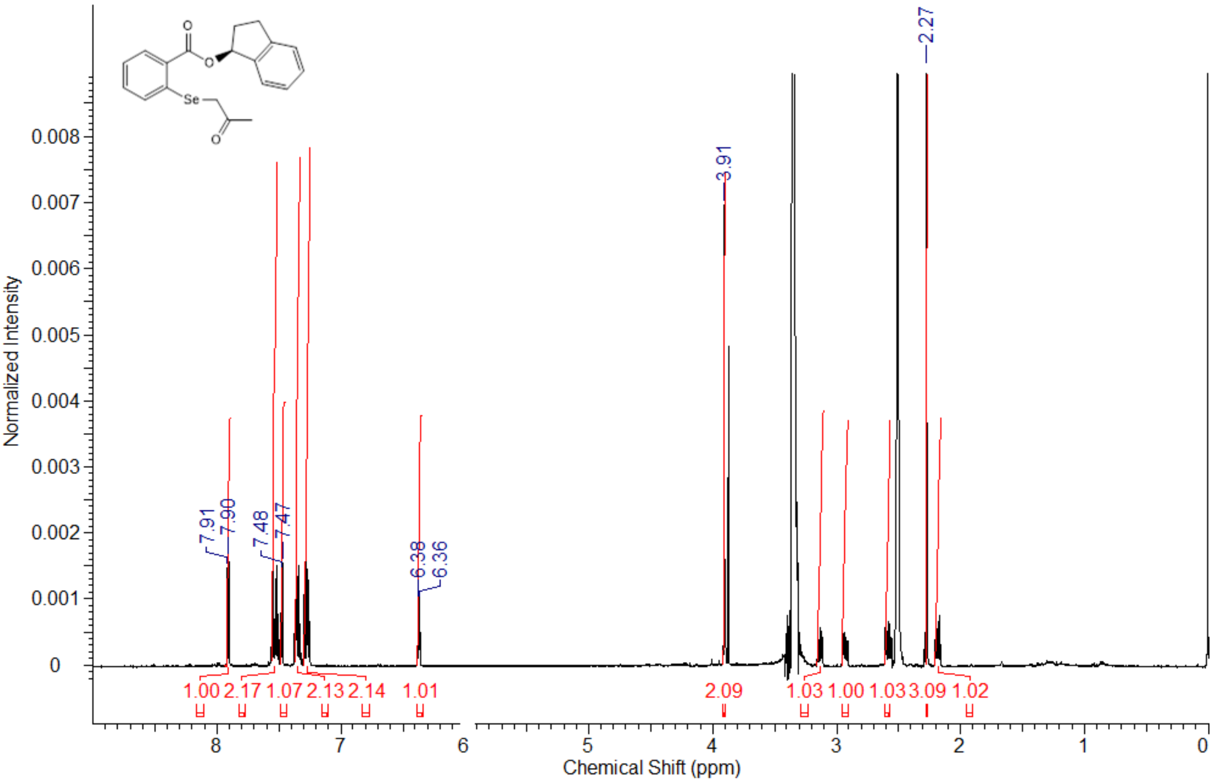

(a)

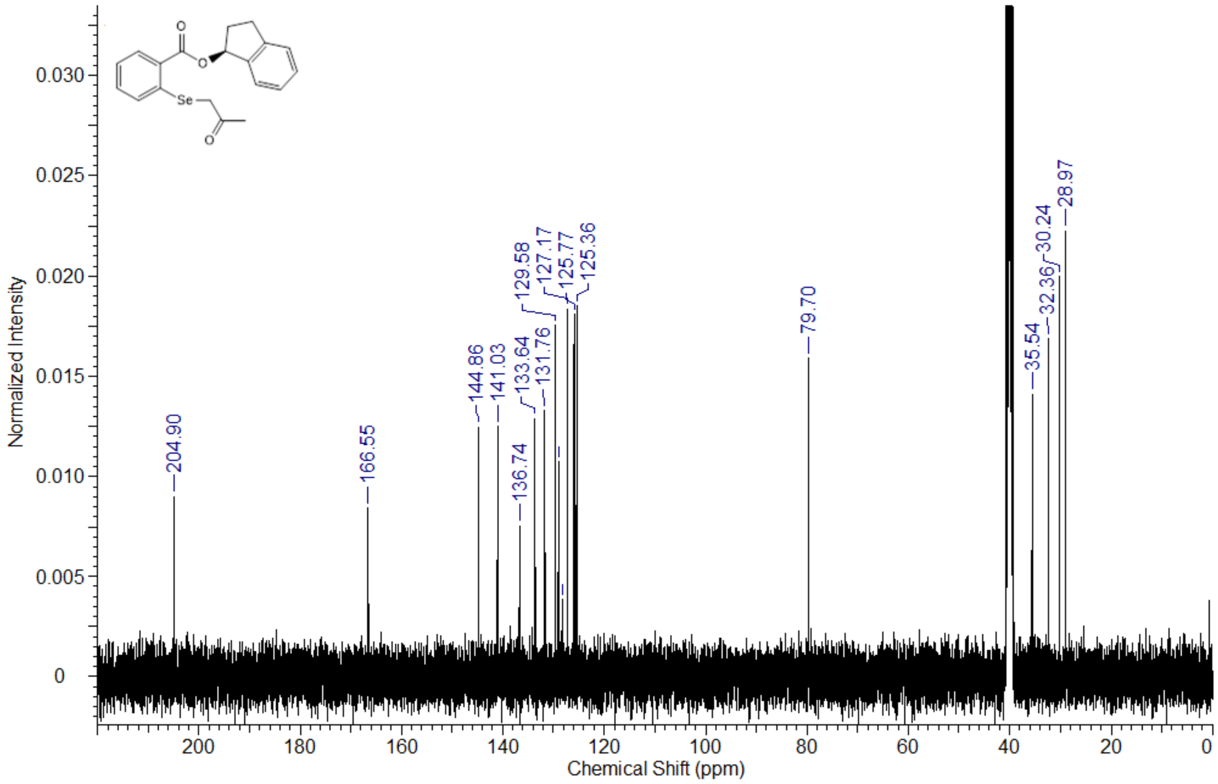

(b)

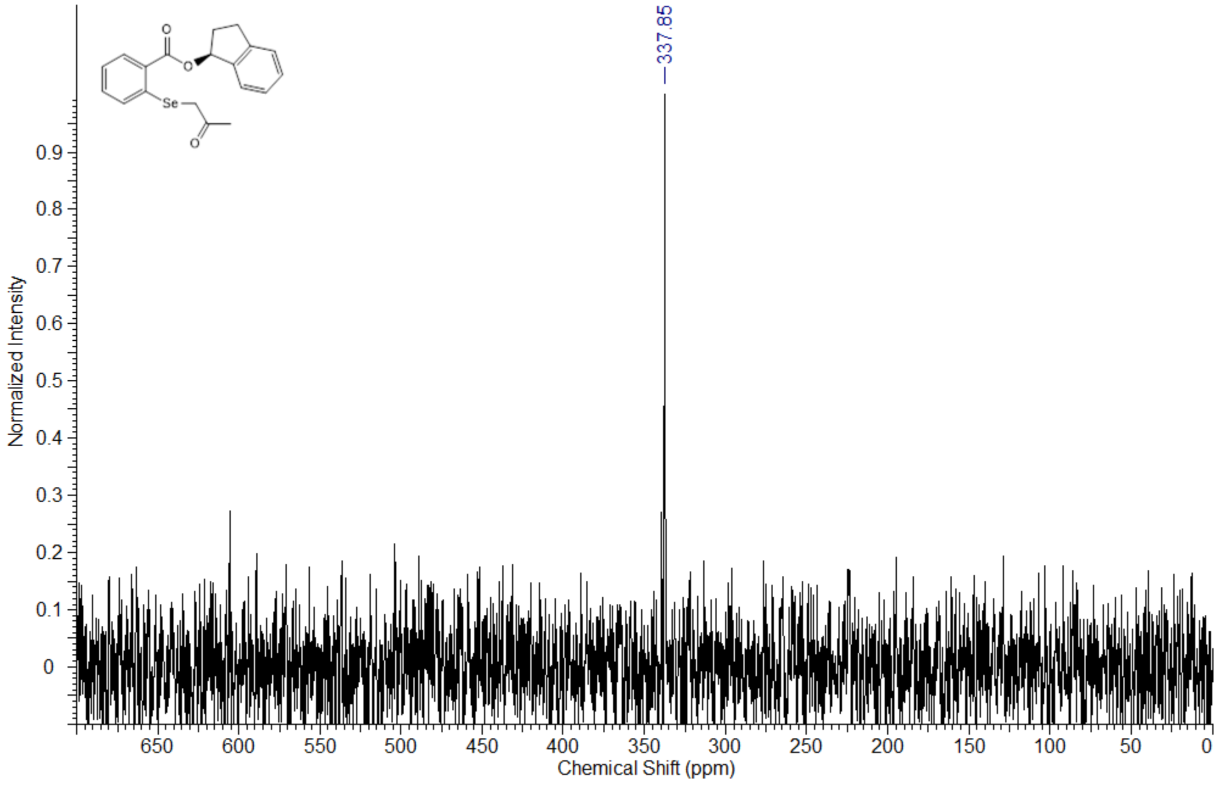

(c)

Figure S14. (**a**) 1H NMR, (**b**) 13C NMR, and (**c**) 77Se NMR spectra of *O*-((*S*)-(+)-2,3-dihydro-(1*H)*-inden-1-yl)-2-((2-oxopropyl)selanyl)benzoate **24**

2. Antioxidant activity measurement

Table S1. Results of antioxidant activity measurement of integration from 1H NMR spectra after reaction time 5 min and 15 min for all compounds

Compound	5 min			15 min		
	Integration DTT^{red}	Integration DTT^{ox}	**Remaining DTT^{red}[%]**	Integration DTT^{red}	Integral DTT^{ox}	**Remaining DTT^{red}[%]**
**12**	21.20	1	95.50	21.20	1	95.50
**12**	19.57	1	95.14	18.31	1	94.82
**13**	29.59	1	96.73	28.11	1	96.56
**13**	28.51	1	96.61	25.17	1	96.18
**14**	13.01	1	92.86	7.90	1	88.76
**14**	12.57	1	92.63	7.46	1	88.18
**15**	14.90	1	93.71	13.80	1	93.24
**15**	14.13	1	93.39	13.21	1	92.96
**16**	19.85	1	95.20	19.00	1	95.00
**16**	18.69	1	94.92	18.03	1	94.75
**17**	10.20	1	91.07	9.90	1	90.83
**17**	9.64	1	90.60	9.30	1	90.29
**18**	14.53	1	93.56	11.00	1	91.67
**18**	14.05	1	93.36	10.51	1	91.31
**19**	20.00	1	95.24	19.00	1	95.00
**19**	19.10	1	95.02	18.30	1	94.82
**20/21**	23.27	1	95.88	16.22	1	94.19
**20/21**	22.72	1	95.78	17.54	1	94.61
**10/22**	24.86	1	96.13	22.07	1	95.67
**10/22**	25.01	1	96.16	22.37	1	95.72
**23/24**	20.40	1	95.33	17.8	1	94.68
**23/24**	14.40	1	93.51	13.4	1	93.06

Table S2. Results of antioxidant activity measurement of integration from 1H NMR spectra after reaction time 30 min and 60 min for all compounds

Compound	30 min			60 min		
	Integration DTT^{red}	Integration DTT^{ox}	**Remaining DTT^{red}[%]**	Integration DTT^{red}	Integral DTT^{ox}	**Remaining DTT^{red}[%]**
**12**	19.56	1	95.14	18.07	1	94.76
**12**	16.95	1	94.43	15.72	1	94.02
**13**	18.76	1	94.94	17.61	1	94.63
**13**	19.52	1	95.13	15.43	1	93.91
**14**	5.20	1	83.87	3.3	1	76.74
**14**	4.01	1	80.04	2.92	1	74.49
**15**	13.50	1	93.10	12.7	1	92.70
**15**	12.87	1	92.79	12.16	1	92.40
**16**	18.10	1	94.76	17.7	1	94.65

**16**	17.45	1	94.58	16.3	1	94.22
**17**	9.70	1	90.65	8.5	1	89.47
**17**	9.00	1	90.00	8.00	1	88.89
**18**	8.50	1	89.47	6.40	1	86.49
**18**	8.01	1	88.90	5.74	1	85.16
**19**	18.00	1	94.74	17.00	1	94.44
**19**	17.20	1	94.51	16.10	1	94.15
**20/21**	21.00	1	95.45	18.15	1	94.78
**20/21**	12.33	1	92.50	8.48	1	89.45
**10/22**	20.57	1	95.36	20.01	1	95.24
**10/22**	24.01	1	96.00	17.16	1	94.49
**23/24**	16.58	1	94.31	13.70	1	93.20
**23/24**	13.00	1	92.86	12.00	1	92.31

3. DPPH Radical Scavenging Assay

The calibration curves were created by increasing volumes of tested compounds. Solutions in methanol to a 0.5 mL methanolic DPPH radical (0.3 mM) made up with methanol to the 2.0 mL. All solutions were measured in triplicate against a reagent blank (2 mL of methanol + 0.5 mL of DPPH methanolic solution) after 15 min at 517 nm using a UV-1601 spectrophotometer (Shimadzu, Kyoto, Japan).

The inhibition ratio (%) was obtained from the following equation:

inhibition ratio (%) = $\frac{\mathrm{A1-A0}}{\mathrm{A0}}\cdot$100%, where: A1—absorbance of sample A0—absorbance of the reagent blank.

The 50% DPPH inhibition (IC_{50}) was calculated by the linear regression analysis between the radical scavenging percentage against the tested compound concentration. Finally, the results of DPPH radical scavenging activity by β-carbonyl selenides were presented as Trolox equivalent antioxidant capacity (TEAC) and calculated as follows:

$$\mathrm{TEAC} = \frac{\mathrm{Trolox\ IC\ 50\ [mM]}}{\mathrm{tested\ compounds\ IC\ 50\ [mM]}}$$

Table S3. The results of DPPH Radical Scavenging Assay

No	**12**	**13**	**14**	**15**	**16**	**17**
IC_{50} [mM]	2.7689	1.2762	1.5493	1.2229	0.6397	2.0772
±SD	± 0.1019	±0.0132	±0.1285	±0.0074	±0.0034	±0.0558
No	**18**	**19**	**20/21**	**10/22**	**23/24**	**Trolox**
IC_{50} [mM]	4.4931	0.0933	0.1254	3.3500	0.6730	0.0740
±SD	±0.0072	±0.0023	±0.0049	±0.0068	±0.0182	±0.0021

Where: SD - standard deviations
